# Stone axes throw new light on Baltic stone age mortuary rites

**DOI:** 10.1038/s41598-024-66854-9

**Published:** 2024-07-13

**Authors:** Anđa Petrović, Aija Macāne, Ivars Strautnieks, Laimdota Kalniņa, Elisabeth Holmqvist, Emily M. Hunter, Diederik Pomstra, Helen Goodchild, Ana Harto Villén, Ilga Zagorska, Mark Edmonds, Kerkko Nordqvist, Aimée Little

**Affiliations:** 1 Department of Archaeology, Faculty of Philosophy, University of Belgrade, Čika Ljubina 18-20, Belgrade, 11000 Serbia; 2 Department of Cultures, University of Helsinki, Unioninkatu 38F, 00014 Helsinki, Finland; 3 Institute of Latvian History, University of Latvia, Kalpaka bulvāris 4, Riga, 1050 Latvia; 4 Faculty of Geography and Earth Sciences, University of Latvia, Jelgavas iela 1, Riga, 1004 Latvia; 5 Centre for Anatomical and Human Sciences, Hull York Medical School, Heslington, York, YO10 5DD UK; 6 Faculty of Archaeology, Leiden University, Einsteinweg 2, 2333 CC Leiden, The Netherlands; 7 Department of Archaeology, University of York, King’s Manor, Exhibition Square, York, YO1 7EP UK; 8 Independent Researcher, Madrid, Spain; 9 Helsinki Collegium for Advanced Studies, University of Helsinki, Fabianinkatu 24, 00014 Helsinki, Finland; 10 Department of Archaeology, Centre for Artefacts and Materials Analysis, University of York, PalaeoHub, Wentworth Way, York, YO10 5DD UK

**Keywords:** Archaeology, Geochemistry

## Abstract

Despite their ubiquity, Mesolithic lithic tools given as funerary offerings have rarely been studied in detail. Whereas personal ornaments (e.g. beads, pendants) are commonly interpreted as markers of social identity and status, archaeologists have struggled to understand the stone tools, commonly regarded as “utilitarian” items. As a result, this class of grave goods has not received the same level of attention, leaving a significant gap in our understanding of Mesolithic mortuary behaviours. Our research challenges long-lasting perceptions of lithic tools as strictly utilitarian objects and draws on studies of one of the most substantial stone axe funerary collections from one of the largest Stone Age cemeteries in Europe–Zvejnieki, Latvia. Evidence suggests the selection of unused axes as grave offerings, while unusual wear traces on an axe found in a female grave (no 57) raises questions about its use in the burial rites. Using a multi-proxy approach, we compare life histories of axes placed in burials to those recovered from contemporary, nearby settlement contexts. Finally, a strong correlation between axes and women and children at Zvejnieki challenges gendered stereotypes of stone tools, historically regarded as possessions of the adult male members of Stone Age societies.

## Introduction

Flaked and/or ground stone tools are ubiquitous on Mesolithic sites across northern Europe. That may be why we often treat them as utilitarian items—tools that mattered only in terms of their immediate function. Nowhere is this clearer than in research on artefacts found in Mesolithic mortuary contexts. While beads, pendants and other items are routinely considered as symbols of identity, status and cultural customs^1–3^, lithic artefacts are frequently overlooked. This tendency persists despite evidence that some stone tools had complex life histories prior to entering the grave^4–7^. At a broad temporal and spatial scale, the inclusion of axes in funerary assemblages is a Northern European cultural tradition, with examples reported from Ireland in the west to Russia in the east^5,8,9^.

Here we report on new scientific analysis of one of the largest assemblages of stone axes from European Stone Age funerary contexts (Supplementary Table S1)^10,11^. The burial site is Zvejnieki in Latvia, itself one of the largest Stone Age burial grounds in Europe and is one of a small number of hunter-gatherer-fisher burial grounds with a relatively high number of axes. Examples exist of similar size axe grave good assemblages from Stone Age cemeteries, e.g. Dudka in Poland^12,13^, Skateholm in Sweden^14^ and Brevenniy cemetery in Russia^15^; however, what makes Zvejnieki unique is the large amount of stone axes from the associated settlement areas. These broadly contemporary settlement contexts containing axes provide an important comparative setting in which to assess evidence for “special treatment” of those found in the mortuary contexts.

Here we present an in-depth analysis of the axe found in burial 57. This axe, which displays unusual wear traces, was found in the grave of an adult female, regarded as having one of the “richest” burial assemblages at Zvejnieki. New evidence for the possible utilisation of the axe from burial 57 within the female’s mortuary rites is presented. To contextualise the results of our analysis of the axe from burial 57, we present comparative results from analyses conducted on the four other axes found in Zvejnieki burials. Using an integrated methodological approach, combining new geological, technological and microwear analysis, alongside existing spatial, contextual and osteological data, we further investigate differences between mortuary and domestic use/treatment of stone axes at Zvejnieki. Local production of axes is evidenced, with hints of movement of material and/or people particularly during the site's later stages of occupation. Our research reveals a pattern whereby axes only occur in the burials of women and women with children, even though adult females make up just 15% of the sexed individuals buried at Zvejnieki^16^.

### Zvejnieki—site location and history of research

**Figure 1:**
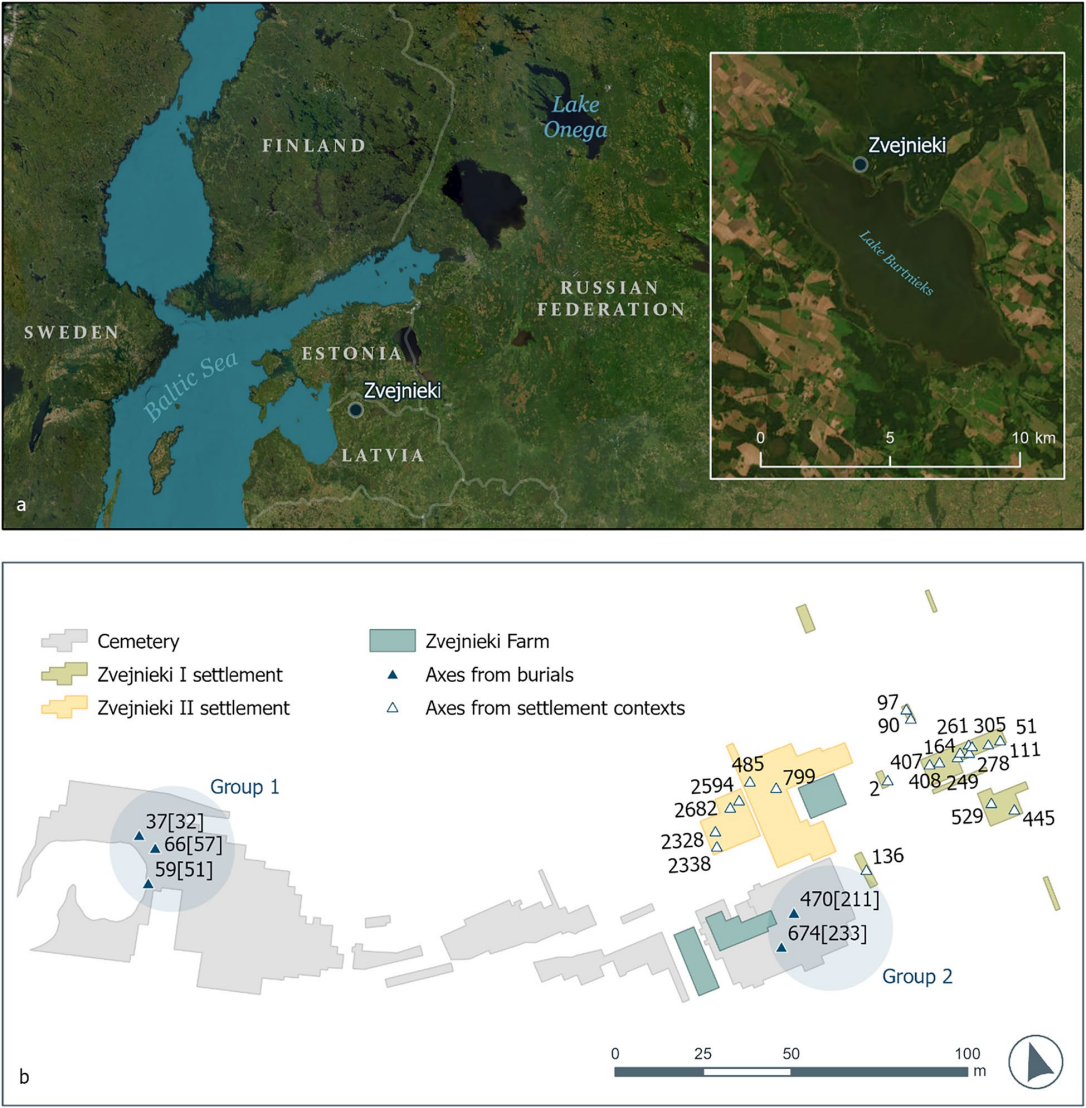
(**a**) Map showing the location of Zvejnieki, Latvia and Lake Onega, Karelia (Data © Estonian Land Board, Latvian Geospatial Information Agency, State Land Service of Latvia, Earthstar Geographics) and (**b**) spatial distribution of burial axes and axes sub-sampled from settlement contexts; the find numbers given refer to the collections of the National History Museum of Latvia (main collection number VI92:, VI168: for settlement and VI93: for burial finds). Only the suffix number is shown here, see Table 1 and Supplementary Table S1 for full finds numbers of the burial and settlement axes). Maps produced by Helen Goodchild using ArcGIS Pro 3.1.3, which is the intellectual property of Esri (www.esri.com) and is used herein under license. Copyright © Esri. All rights reserved.

The Zvejnieki settlement and burial site lies on the northern shore of Lake Burtnieks in northern Latvia (Fig. 1a). It was discovered during gravel extraction and was extensively studied in the 1960s and the 1970s, with additional investigations in the early 2000s^10,11^. Over 330 burials containing the remains of at least 350 individuals have been discovered^10,16^. Grave goods may or may not be present and often include various objects of antler, bone, and animal teeth and, particularly in the later part of the site’s use, lithic artefacts and amber. The settlement area has produced material typical for the Eastern Baltic Stone Age: cultural layers contain storage pits, possible dwelling remains and fireplaces, as well as thousands of lithic (mostly flint), bone and antler artefacts and pottery sherds and an abundant refuse fauna. Radiocarbon dates place the cemetery’s use between 7500 and 2500 cal. BC, in addition to some more recent burials^17,18^, making Zvejnieki the longest-used Stone Age cemetery in northern Europe. The radiocarbon dating of the settlement site is so far based on only two–three dates, but based on these and the artefact typology, the use of settlement even exceeds the use of cemetery and may have begun as early as the 9th millennium cal. BC^11^. In the periodisation followed in north-eastern Europe, Zvejnieki belongs both to the Mesolithic and the Neolithic. The vast settlement site has been roughly divided typologically into upper Mesolithic (Zvejnieki II) and lower Neolithic (Zvejnieki I). In this region, the boundary between periods is set by the advent of pottery technology, which took place in Latvia in the second half of the 6th millennium cal. BC. Farming only began to spread in the area in the 3rd and 2nd millennia cal. BC, so our main period of interest was characterised by hunter-fisher-gatherer subsistence strategies. For this reason, the term “Stone Age” will be used here, in that it avoids a misleading emphasis on a change in lifeways, as seen in many other parts of Europe^19^.

## Results

### Axes from Zvejnieki funerary and domestic contexts

A total of five axes from burials at Zvejnieki were examined in the National History Museum of Latvia. Of the 330 burials, only 5 burials (1.5%) contain axes. While this number is significantly lower than most other types of grave goods, such as tooth pendants or amber, our research represents the entire assemblage of axes from Zvejnieki and is—to the best of our knowledge—the only, multidisciplinary study focussed on the use and deposition of stone axes in European hunter-gatherer-fisher burials. At Zvejnieki, axes occur singularly in graves. Three belong to females, with burial 211 an adolescent female of 14–19 years. The two children were part of multiple burials, in both cases placed with an adult female individual (Table 1; see also^10^). To date, aDNA sexing of human remains from Zvejnieki has only been undertaken on 24 individuals^20^; unfortunately, this does not include the five with axes. It is worth noting, however, that the DNA based sex identifications are consistent with the earlier osteological sex estimations^10,21^.

**Table 1:** Context and association of axes with human remains in burials.

Group	Burial No	Find No	Burial type	Age	Sex	Location of axe in relation to body
1	32	VI93:37	Multiple	0–7	Indet	Chest
	51	VI93:59	Multiple	Infans	Indet	Feet
	57	VI93:66	Individual	Adult	Female	Head
2	211	VI93:470	Individual	14–19*	Female**	Indeterminate (disturbed context)
	233	VI93:674	Individual	Adult	Female	Pelvis

Group 1 and 2 refers to burial axes found in close spatial proximity. Age and sex after Zagorskis 2004^21^.

*Age of the individual is given as 18–25 in Zagorskis 1987^10^ but is 14–19 in Zagorskis 2004^21^.

**Sex of the individual was originally classified as male in Zagorskis 1987^10^ but in Zagorskis 2004^21^ is sexed as female.

Compared to the burials, information on the depositional context of axes recovered from the Zvejnieki settlement is more generic (i.e. ‘cultural layer’). This is in part because these excavations have yet to be published, but also because of the mixed, multi-period contexts and relatively large recovery units used. In total, 21 settlement axes were analysed for this study, which account for around half of the recognisable stone axes in the settlement contexts. The selection was made according to preservation: only sufficiently intact items with recognisable morphological features were included and of these only the 11 best preserved items were analysed microscopically for wear traces.

### Spatial and temporal distribution of axes from funerary contexts

Three axes (burials 32, 51, 57) were found in proximity in the NW part of the cemetery (group 1), while the other two (burials 211, 233) are similarly near each other in the SE part (group 2), about 150 m away (Table 1; Fig. 1b). Burials in group 1 contained red ochre, while group 2 burials were filled with black soil. All burials included varying amounts of animal tooth pendants; burials 57 and 211 also contained other modified bone items and worked flint^16,21^.

Only burial 57 has been radiocarbon dated: a skull fragment gave a date of 6825 ± 60 BP (Ua-3636^22^), recently reservoir-corrected to 5750–5250 cal. BC^23^. Due to the similar burial customs and morphology of the axes in group 1 and the tooth pendants, which consist only of herbivore teeth, a tentative dating to the 6th millennium cal. BC can also be proposed for burials 32 and 51. The burials of group 2 likely date to the 4th millennium cal. BC based on the grave goods (burial 211: large bifacial flint artefacts, burial 233: axe morphologically resembling a miniature version of a so-called East or Russian Karelian type and carnivore tooth pendants). Even if the spatial placement of burials does not always work as a dating criterion at Zvejnieki^16^, it seems to be relevant here. Burials within group 1 and 2 are in spatial vicinity, but with group 1 and 2 located far apart from each other. Their internal contemporaneity is supported by similar burial customs and typologies of the grave goods. Due to a lack of radiocarbon data, it is not possible to verify the contemporaneousness of the settlement phases and burials beyond broad typochronological horizons; this is a common situation in the research area, where the total number of 14C determinations is generally low.

Of the burials with axes, only burial 57 was anatomically complete. While the rest of the burials with axes were disturbed by later burial activities (as is common at Zvejnieki), there does not appear to be any spatial pattern regarding the positioning of the axes in relation to the body; they were found in varying locations between the legs and the head (see Table 1).

### Burial 57

**Figure 2:**
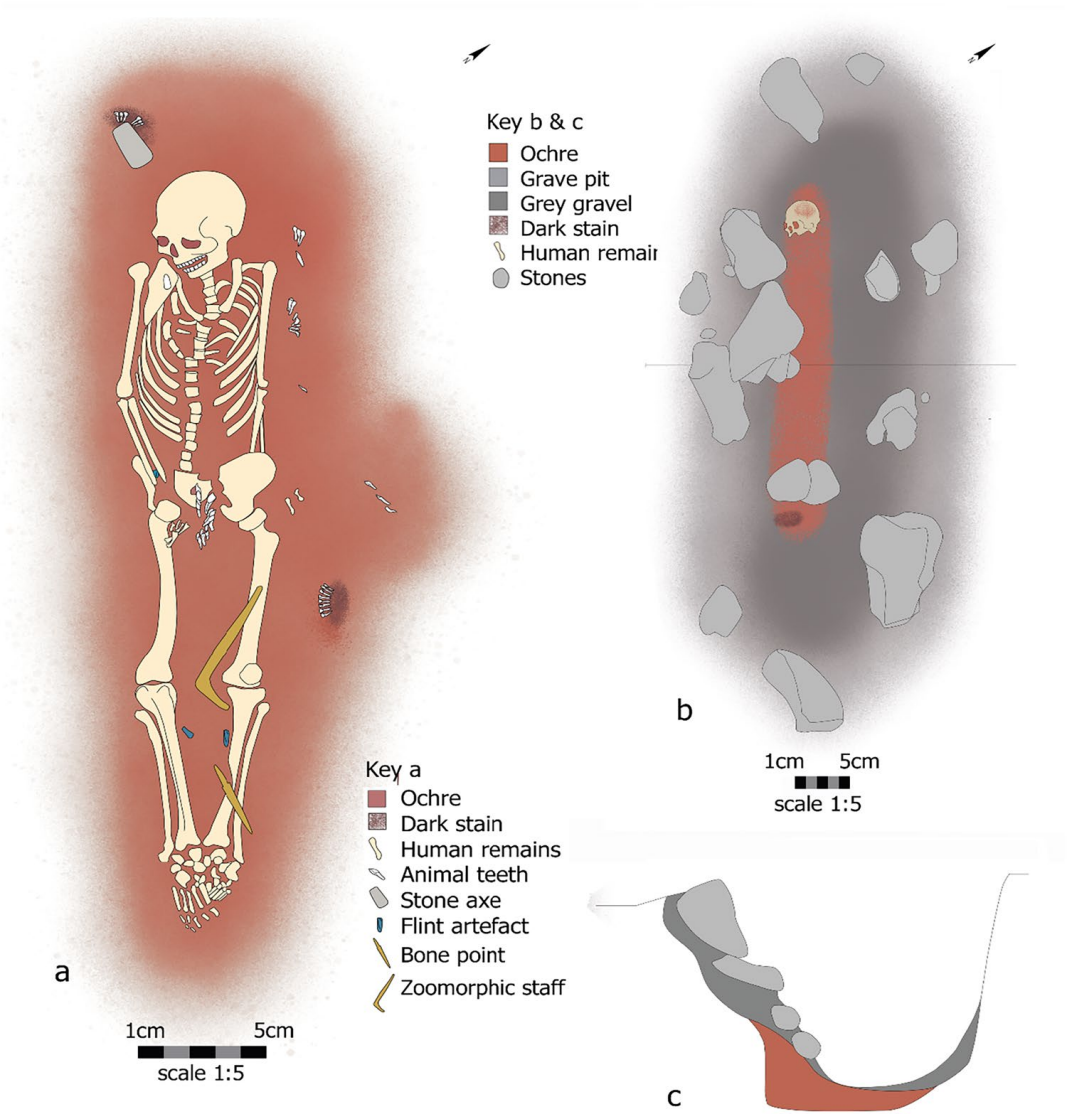
Schematic plan of burial 57 (**a**) Composition of burial 57, showing the spatial relationship between the adult female’s skeletal remains and the different items recovered from her grave. This includes a reconstruction of the spatial arrangement of two separate clusters of red deer teeth and axe VI93:66, as they were recorded during excavation. Stone setting (**b**) above the burial 57 showing the distribution of ochre staining. Profile of the grave pit (**c**) containing stones related to the grave construction. Illustration by Hege Vatnaland, based on drawings by I. Ārmane (Institute of Latvian History, University of Latvia).

Burial 57 (Fig. 2a) contained the remains of a female adult placed in extended supine position with head oriented to the NW, together with an intense ochre layer surrounding the skeleton^21^. A stone setting covered the burial (Fig. 2b), with stones related to the grave construction visible in the grave profile (Fig. 2c). Such stone settings are known from a small number of other Zvejnieki burials^10^ The fill of the burial contained grey gravel and stones. Six unmodified red deer (*Cervus elaphus*) incisors, sourced from a single animal, were found partially underlying the axe to the right and just above the female’s head^10,16^. A dark area underlying and surrounding the axe and teeth can be seen in the original site illustrations and may represent staining from decayed organic matter. A similar arrangement of permanent red deer front teeth, with similar wear traces visible on the lingual side of all teeth—indicating they too were sourced from one animal and re-arranged in anatomical order—were found to the left of the female’s leg area^16^. Like the axe-pendant arrangement by the head, these teeth were associated with an area of dark staining. In both cases, it is possible that the teeth were attached to an organic pouch or similar, with the staining representing the degraded pouch.

In addition to the teeth and axe, other offerings were also found, including a zoomorphic staff of bone and two blades, a backed blade, and a scraper of flint^10,24^. Combined, the diversity, quantity and uniqueness of this grave assemblage has led to this burial being interpreted as one of the “richest” at Zvejnieki.

### Archaeological wear traces on the axe from burial 57

**Figure 3:**
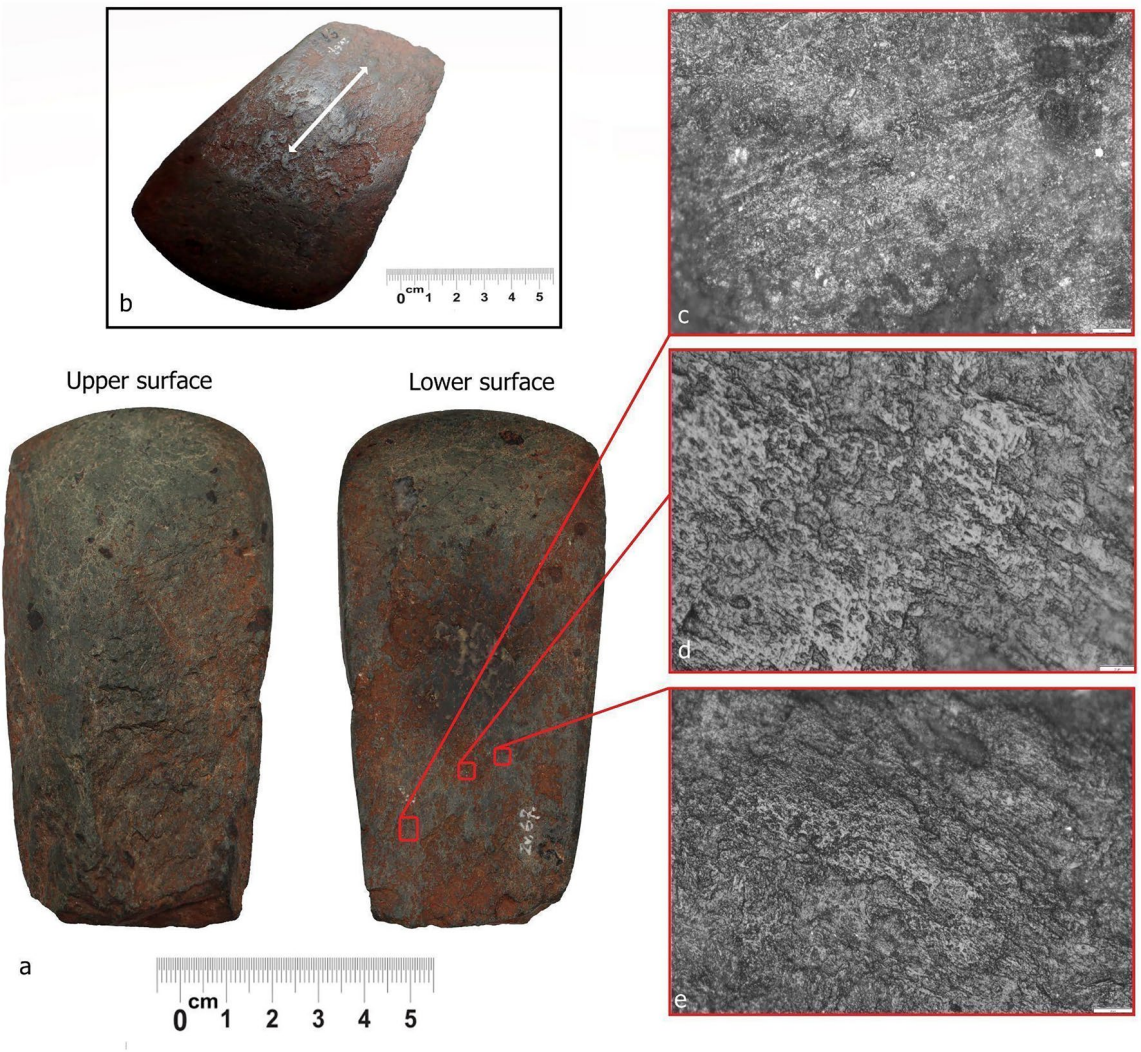
Archaeological macro and microwear traces on axe VI93:66 from burial 57 (**a**) Upper (slightly convex) and lower (flat) surface of the axe, (**b**) Macroscopically visible wear traces resulting from use on the lower surface; the arrow indicates the longitudinal orientation of the traces (parallel with the long axis of the axe) and directionality of the activity (rubbing motion), (**c**) Micrograph of polish with linear directionality and a rough texture with an open to half-tight linkage resembling working of mineral material, scale bar equal to 50 μm, (**d**) Micrograph showing a mixture of worked materials and resulting polishes, represented by smooth texture, slightly rounded topography indicating working semi-dry hide with a fatty additive, scale bar equal to 20 μm, (**e**) Micrograph of more developed areas of combined polish, dominated by smooth and rough texture and tight to covered linkage, associated with processing of animal (semi-dry hide/fat) and mineral (ochre) materials, scale bar equal to 50 μm.

Microwear analysis at high power magnification revealed no visible signs of use of the blade edge of the axe from burial 57, which is where use traces (other than those derived from hafting) would usually occur. One of the axe surfaces does, however, display visible traces of use (Fig. 3a). The lower, flat surface is well-polished with clear directionality of working: a back–forth grinding motion, with traces running in parallel with the long axis of the axe (Fig. 3b–c). Ochre is concentrated on this lower flat surface and has penetrated deeply; its location, distribution and quantity indicate working of this mineral. By comparison, the upper convex surface displayed only superficial traces of ochre, likely emanating from the burial environment.

The lower flat surface displays two different types of polish. The first is a more general one, created on the less developed, lower areas, and is characterised by rough to smooth texture, granular topography with open to half-tight linkage. The rougher texture resembles polish derived from working minerals^25,26^, (Fig. 3c). The second type is more developed. This latter polish is bright with smooth texture, flat topography, and is more linked-up, resulting from the processing of soft-medium animal material, possibly semi-dry hide with a fatty additive (Fig. 3d–e). It is unlikely that the wear traces have resulted from containment and curation within the organic pouch as the wear is restricted to just the lower axe surface.

### Experimental archaeological results

**Figure 4:**
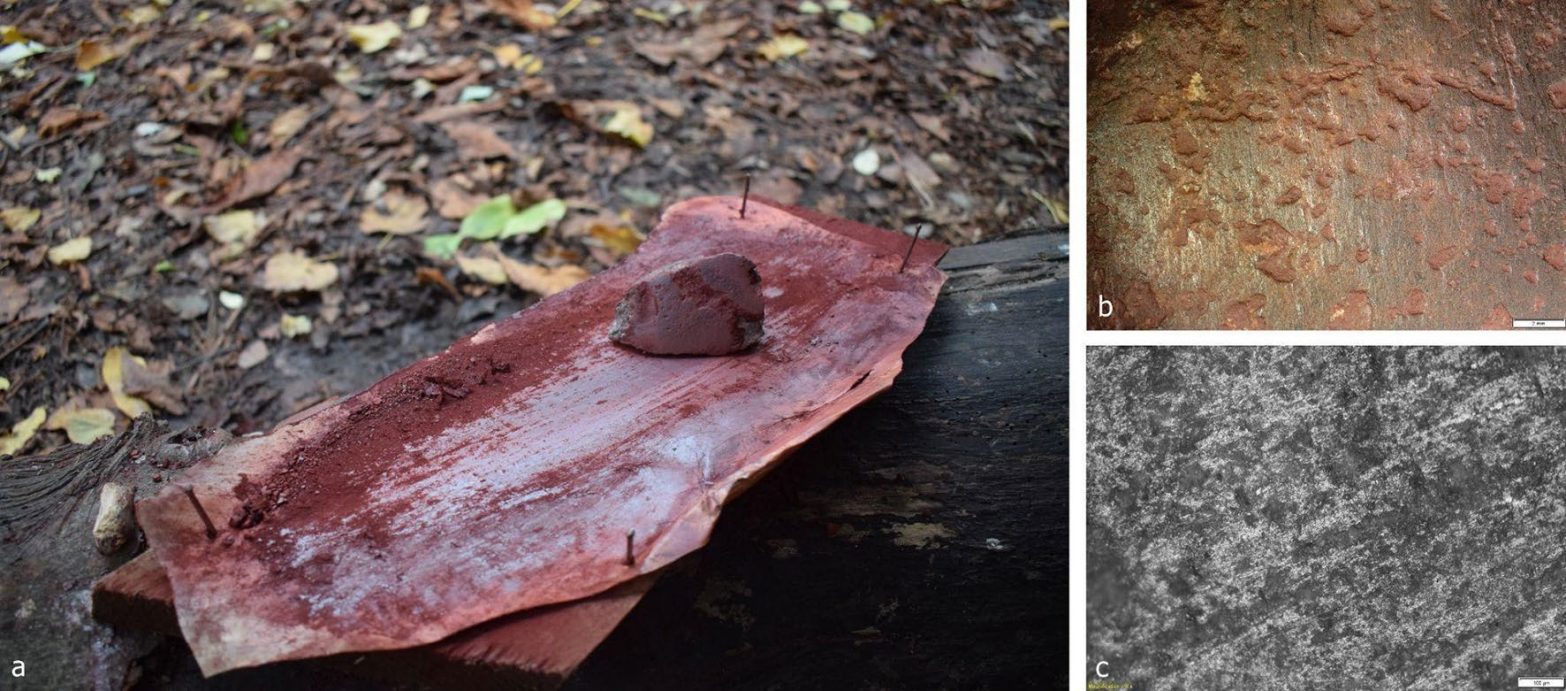
(**a**) Experimental polished stone surface used for grinding ochre and lard on rawhide supported by wood, (**b**) Macro traces after 30 min of back and forth rubbing activity, scale bar equal to 2 mm, (**c**) Micro traces after 60 min of back and forth rubbing activity, scale bar equal to 100 μm.

To replicate the archaeological wear traces recorded on the axe from burial 57, we carried out eleven experiments, which generated different wear traces for comparison with the archaeological samples (Supplementary Table S2). The experimental polished stone surface (A9, Supplementary Table S2) that produced the most comparable wear traces to those found on the axe from burial 57 was used for rubbing ochre (already ground into powder) and lard on rawhide positioned on a wooden surface (Fig. 4a). Macro traces were represented by lines parallel to the longer edge of the polished surface, which became very glossy by the end of its use (60 min), (Fig. 4b). These observations were visible with the naked eye and low power magnification (0.8–1x). Micro traces visible across the worked surface could be divided into two types. Less developed surfaces have an open to half-tight linkage and a rougher texture of polish. The most developed areas have flat topography and smooth texture. The directionality of the polish is visible and well-defined (Fig. 4c). The polish, in general, is very bright; however, there are some flat areas consisting of covered linkage and more matte polish. It is possible that this is a result of using lard and ochre as additives. Similarity between archaeological and experimental traces is reflected in the rough to smooth texture of the micro polish together with the areas of flat topography at the most developed localities of the used surfaces.

### Interpretation of wear traces on the axe from burial 57

Considering the compatibility of the archaeological and experimental traces, and the large existing body of functional studies (microwear analysis, experimental archaeology)^27^ it is reasonable to conclude that axe no VI93:66 from burial 57 was possibly used for working ochre mixed with animal fat into hide, with the hide possibly in a semi-fresh state. The motion of use corresponds to a back and forth rubbing activity. In addition, as is best practice in microwear studies^28–30^ photos of the axe and micrographs of wear traces were “blind tested” on a group of six microwear experts at a workshop, Laboratory for Artefact Studies, Leiden University. All agreed that the traces resembled working hide with an ochre additive, in a back/forth motion. Our analysis further established that there are no visible traces of grinding or pecking of lumps of ochre, suggesting that the ochre was probably already in a powdered state when worked into the hide.

It is impossible to know the exact activity and context in which the axe was employed. It might have been used in everyday hide-working activities at the settlement or nearby, prior to its deposition as a grave good. Alternatively, given the funerary context and its unconventional use, with the flat surface of axes (as far as we are aware) not typically used for hide-working, alongside a high concentration of ochre present within the grave, it is possible that was employed in the female’s burial rites.

Because of the funerary context of axe VI93:66 and the material similarity between rawhide and human skin, we could not rule out the possibility that the axe surface had been used to apply a fatty ochre mixture directly on the corpse. However, resulting traces from working a fat and powdered ochre paste in back-forward motion to the skin of one of the co-authors (DP) for 60 min showed limited compatibility (see sample A1 in Supplementary Table S2) with the archaeological wear on axe no VI93:66, making this less-probable. Even though our results show that the axe was used to work a fat and ochre mixture into a piece of rawhide, no such hide is preserved in the burial (or any other burials at Zvejnieki). The site publication^10^ as well as later archaeothanatological studies^31^ did, however, reveal anatomical and taphonomic evidence for wrapping of corpses, including the female from burial 57.

**Figure 5:**
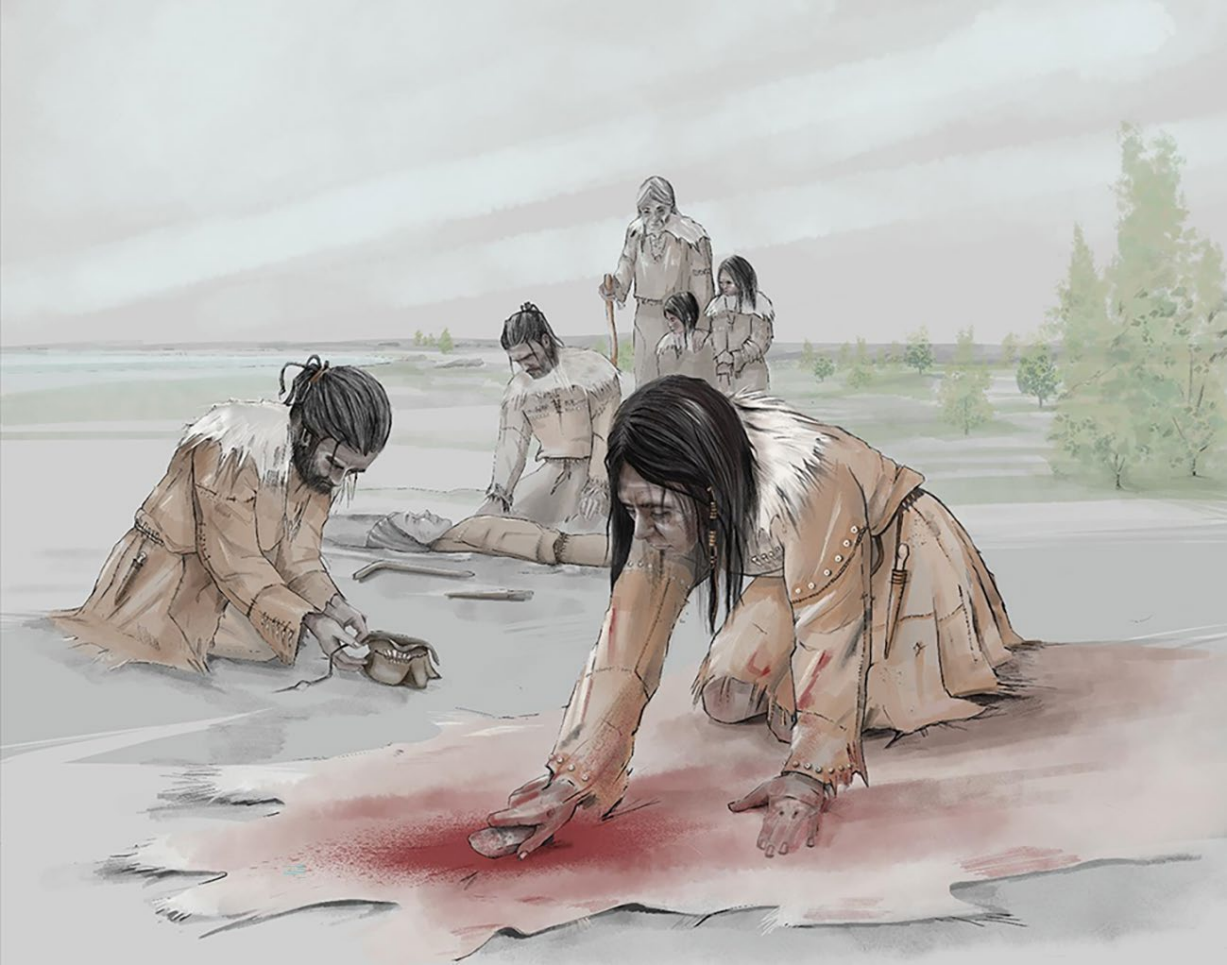
Reconstruction drawing of burial 57, interpreting how the axe may have been used within the female individual’s burial rites. Illustration by Hege Vatnaland.

Given the unusual use of the axe and its depositional context, it is plausible that the traces of ochre and hideworking on its surface resulted from preparing hide used to wrap the female interred in burial 57. After its use, the axe, perhaps because of its association with death, like the female it accompanied, was placed into its own organic container, possibly a hide pouch, long since perished, but evidenced by dark staining of the soil. This pouch may have been decorated with a set of red deer (*Cervus elaphus*) front teeth, extracted from a single animal^16^, with the teeth attached in anatomical order suggesting a degree of intentionality in its making, with the ultimate purpose being the containment of the axe. Given this axe was the only example from either burial or settlement contexts which had been used/treated in this unconventional way, we propose that it may be because of its active role in the funerary rites that it was accorded this special treatment (Fig. 5).

### Microwear analysis of other burial axes and settlement axes

At a macroscopic level, none of the four other axes from funerary contexts displayed conventional signs of usage of the blade edge such as micro-chipping and rounding. Based on these results, we conclude that these four axes had not been used for chopping, cutting or any other activities prior to being placed into the graves. Nonetheless, each axe was analysed at high power (100–200×magnification).

In addition to the axe from burial 57 and the four other axe forms from funerary contexts, a sub-sample of n = 11, approximately a quarter of the total, of axes from the Zvejnieki I and II settlements were analysed microscopically with the same high-power approach (Supplementary Table S3). These 11 settlement axes were selected for microwear based on their preservation and intactness, with some of the other axes from the settlement, including some of those analysed with pXRF, too fragmented from use to analyse microscopically. Microwear analysis of 11 axes from settlement contexts was undertaken to enable a comparison of wear traces and axe usage between domestic and funerary contexts. At a macroscopic level, it was evident that beyond the subsample, only a few of the broader assemblage of settlement axes were complete, with many displaying breakage patterns and heavily damaged edges indicating intensive use to the point of destruction and abandonment. Subsequently, no traces of resharpening or hafting could be observed on any of the eleven axes and, similarly, is unlikely to be visible on the any of the remaining axes from the settlement that were not microscopically analysed. Of the eleven analysed at high power magnification, all showed micro traces of use. These were not always diagnostic, mainly because the traces were poorly preserved or covered in alterations, such as soil sheen. Most blade edges were heavily rounded indicating a prolonged period of use, and in some cases, also the probable processing of harder materials.

Overall, the wear trace evidence clearly points to a difference in treatment between axes utilised in domestic contexts and those recovered from the mortuary sphere. This lack of evidence for use on four of the axes and unconventional traces, which appear connected to funerary activities on the fifth axe from burial 57 (see above), suggests that all axes found in grave contexts were either selected from unused stocks or possibly commissioned as part of the funerary rites.

### Morphology and materials of burial and settlement axes

**Figure 6:**
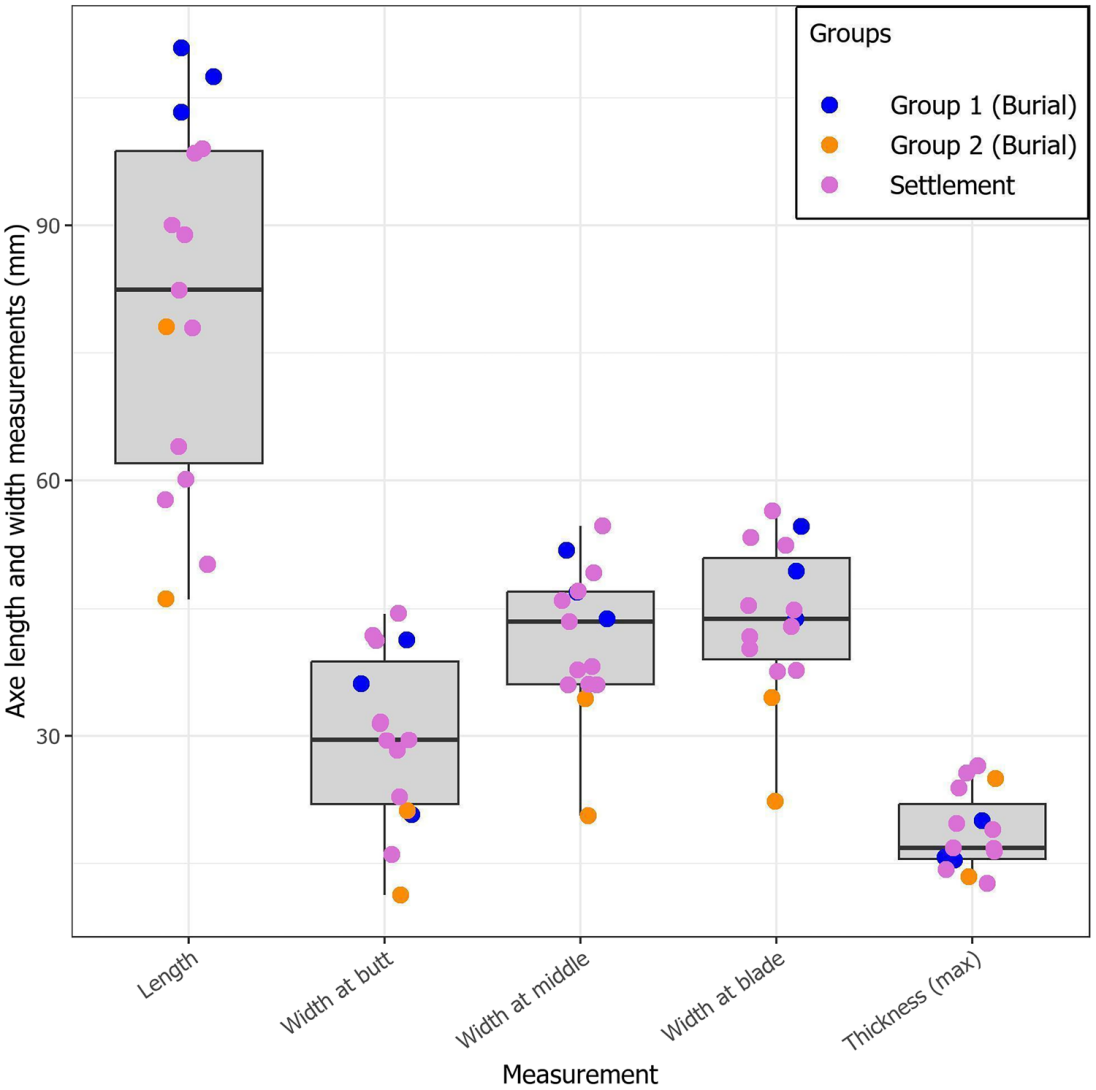
Morphometrics boxplot showing key measurements (mm) for axes (total length, width at three points, and maximum thickness). Axes from group 1 (burial), group 2 (burial) and the settlement are grouped with individual values for each measurement shown as coloured points.

**Figure 7:**
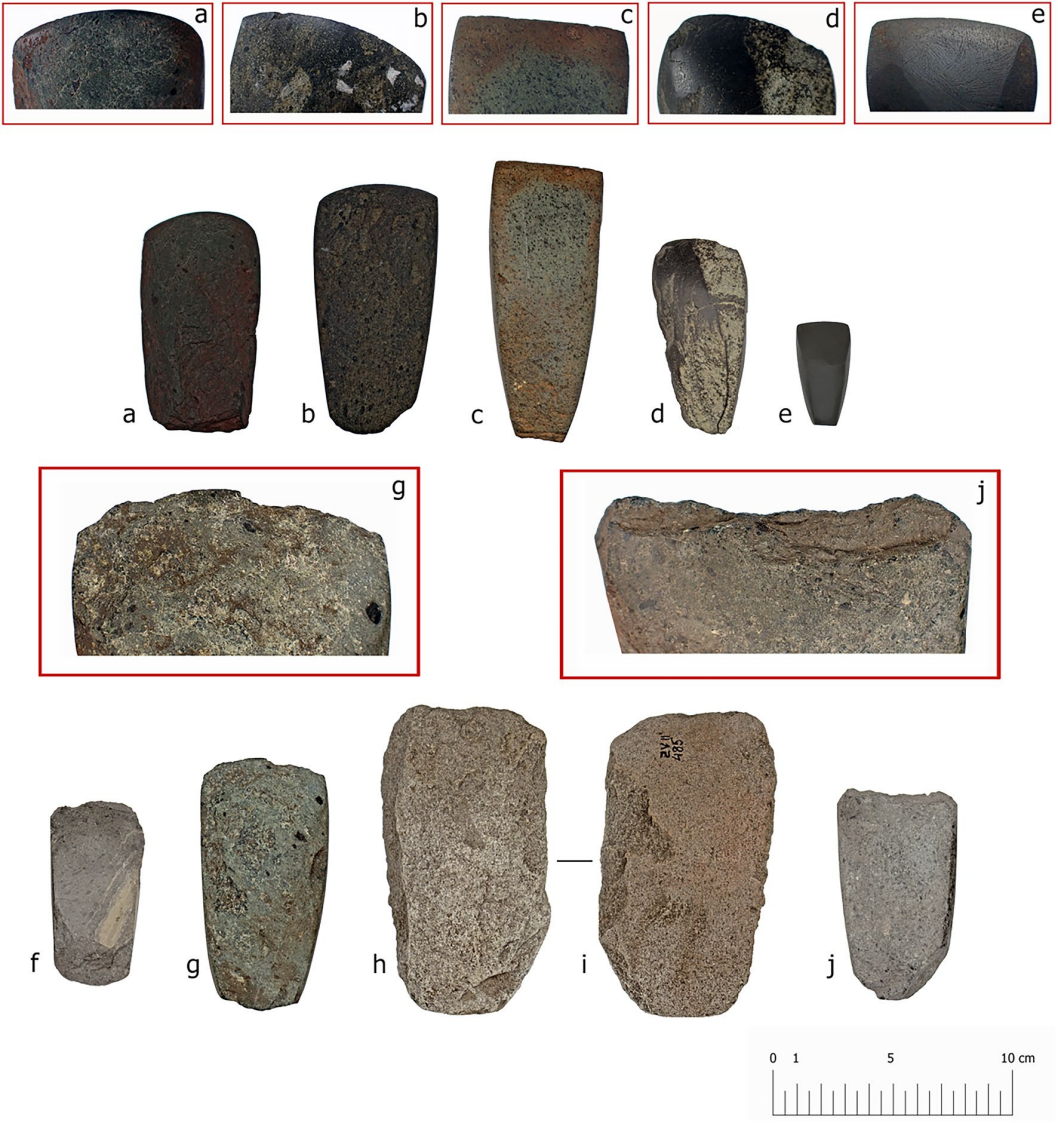
All five (a-e) Zvejnieki burial axes (VI93:66, VI93:59 VI93:37, VI93:470, VI93:674) with (**a**–**c**) included in group 1 and d-e included in group 2. The lower row (**f**–**j**) represents a sub-sample of analysed settlement axes (VI168:2338, VI168:2682, VI168:485 (**h** and **i** show both upper and lower surfaces of VI168:2328), all displaying blade edge damage from use, with insets (**g**, **j**) showing close-ups of the intense degree of use-related damage. Note the difference in condition of the settlement blade edges compared to the burial axes, with the latter intact (insets **a**–**e**).

The axes from burial and settlement contexts at Zvejnieki are broadly typical of the forms found in Mesolithic and Neolithic assemblages throughout the boreal zone^32,33^. Profiles are generally flat; a planar shape widening from the butt to the blade. The majority were initially shaped by flaking/percussion, the final form realised through grinding and polishing to varying degrees. Axes in the group 1 burials are morphologically and technologically similar, all with a length (103–111 mm) greater than those from the group 2 burials and most axes from settlement contexts. The size and integrity of the group 1 axes suggest their relatively contemporaneous manufacture for burial and subsequent deposition; reflecting similar ideas and practices regarding what objects should be included in a grave (Figs. 6 and 7; Supplementary Data S1). Whereas differences between the axes from groups 1 and 2 are chronological.

Based on the results of pXRF and petrographic analysis, we conclude that most of the axes discussed here reflect the use of raw materials that were available in the Burtnieks drumlin field in the Lake Burtnieks area, formed under subglacial conditions (see Supplementary Tables S4 and S5). The one exception to this trend is axe VI93:674 from burial 233. It is made of greenish amphibolite, visually resembling the so-called metatuff found in a restricted area in the western Lake Onega region of Karelia (see Fig. 1a). This raw material was used extensively in its area of origin in the production of widely exported, technologically and typologically very characteristic Russian Karelian artefacts^34^. Since this axe differs from the other Zvejnieki axes but resembles both the raw material and morphology of artefacts from the Lake Onega region, it may well represent an import. Otherwise, sourcing is best characterised as localised and varied, with no clear patterns between geology used and the broadly defined axe types (except those from group 1).

## Discussion

Axes can be regarded as rare grave goods at Zvejnieki, present in just 5 (1.5%) out of the 330 graves excavated; thus, to date, axes have been overshadowed by the other grave offerings. The number of axes and possible axe fragments found in settlement contexts exceeds the amount found in burials by a factor of about 10. Nevertheless, the lack of wear traces on the burial axes makes them strikingly different to the settlement axes. Most of the latter were used to the point of exhaustion, with none displaying wear traces like that from burial 57. This suggests that while axes were recognised as appropriate and important tokens to occasionally place with the dead, their placement was not predicated on prior histories of use and association with particular people. In other words, these were not the specific tools that particular people had carried and used in life. Instead, they may have been sourced from a store of unused axes, including the Karelian-like axe in burial 233, originally possibly exchanged from afar. Another hypothesis, supported by the difference in group 1 burial axe lengths and their undamaged state compared to settlement axes, is that they were made especially for the purpose of accompanying the dead; manufactured for, or as part of, funerary activities.

By combining the different scientific analyses (geology, technology, microwear and experimental archaeology; see Supplementary Information) with other lines of archaeological evidence (spatial, contextual, and other grave good data) from burial 57, we propose the following life history for axe VI93:66. The axe was likely made from a locally sourced stone type (uralite hornblende porphyrite), a glacial cobble, collected from a nearby drumlin. Its maker was possibly familiar with this source and mode of production, with axes made from similar raw materials used as part of everyday domestic activities. Like comparable axe forms placed in nearby burials, the blade edge was unutilised; however, VI93:66 is different to those axes in that its flat lower surface was used prior to deposition. It was used to rub a mixture of ochre and fat into an animal hide in a back-forward motion. While this hide may have then been used to wrap the corpse, with wrapping indicated by archaeothanatological studies, we will never know this for certain. The axe was then deposited, possibly within a decorated pouch. The distinctive deployment of this axe resonates with a growing body of evidence for the use of different types of lithic tools in hunter-gatherer mortuary rituals prior to their deposition as grave goods^4,5,35^.

It is difficult to determine the associations that these axes brought into focus during funerals. However, the patterns that we have challenge some pervasive assumptions. Particularly noteworthy is the association of axes with females and/or children, when only about 15% of the deceased buried in Zvejnieki are determined as adult female (cf. ca. 35% adult male) and approximately 30% are children^10,16^. The association of axes with Mesolithic female burials finds parallels elsewhere, including some of the earliest^36^ and most elaborate^37^ examples. At the very least, this should lead us to question the use of lithic artefacts to sex individuals as male even when sexing is impossible due to young age or poor skeletal preservation^4,38^. Indeed, it is entirely possible that other European burials with axes (and other types of stone tools), which have been assumed to be male are, in fact, female. Moreover, burials of axes with children and infants^38–41^ the provision of “pseudo axes” (unworked stone in the shape of an axe) as grave goods^12,13^ and other ritual deposits^42^ all suggest that axes played both functional and symbolic roles within hunting and gathering societies.

We further suggest that three of the five axes (from burials 32, 51, and 57) belonging to group 1 may have been made and interred more or less contemporaneously during the 6th millennium BC. Of the group 2 axes, a unique axe given to an adult female (burial 233) shares geological and morphological parallels with the East or Russian Karelian tool industry centred in the western Lake Onega region and is unlike any from the Zvejnieki settlement. It can be viewed as a non-local object that was exchanged over long distances before being deposited in a burial at Zvejnieki—together with an also unique composition of other grave goods consisting only of dog tooth pendants^16^. The axe also reflects changes observed in hunter-gatherer genetic ancestry^20,43^ and provides additional evidence of the movement of people and increasing connections north and northeast during the final stages of the site’s use during the 4th millennium BC.

Combined, the long-distance Karelian example, deposited unused (burial 233), the locally sourced unused axes (burials 32, 51, 211) and the unusual use/treatment of the locally made axe (burial 57) help shed new light on the role of axes in Stone Age Baltic Europe. Such insights are vital not just for understanding what grave goods symbolise but the significance of axes more broadly within hunter-gatherer worldviews. Not only did they provide practical aid with everyday tasks, but they were also incorporated (albeit only occasionally) into mortuary activities. Finally, whilst we may never know exactly who used the settlement axes in everyday tasks, what is clear is that contrary to gendered stereotypes, it was a small number of women and children at Zvejnieki who received axe forms at death.

## Methods

### Microwear methodology

Microwear analysis of both archaeological and experimental samples were conducted implementing a low and high-power approach^25,44–48^. Low-power approach (< 100×magnification) consists of using the stereomicroscope to perform the preliminary analysis and to describe the macro traces, while high-power approach (100–400×magnification) is focused on the identification of the micro polish and interpretation of the worked material. For the purposes of this study two stereomicroscopes were used: Olympus SZ61 together with Olympus LC30 camera and BRESSER Science ETD-201. Micro analysis was done with metallographic microscope Olympus BX53M with Olympus DP74 and Olympus SC180 cameras. Cleaning of the archaeological samples was carried out with ethanol, while experimental polished tools were also cleaned with the detergent in an ultrasonic tank for 15 min and then rinsed.

### Experimental archaeology

To test the results of the microwear analysis, an actualistic experimental programme was designed. In total, 11 polished working surfaces were produced, resulting in a reference collection of axe-like surfaces that were used in ochre grinding/crushing, on different material substrates of varying hardness.

Three different types of metamorphic rocks were chosen for the making of the polished surfaces: Porphyritic Trachyte, Hornfels and Cordierite Hornfels, all purchased commercially. Unfortunately, it was not possible to use the exact same raw materials as used for axes at Zvejnieki because of logistical constraints. However, the replica axe-surface geology was of comparable porosity and hardness, providing an appropriate proxy. Axe-like surfaces were produced by polishing the natural flat facets of the rocks that could be used in the trials. Based on the microwear analysis, which revealed contact with mineral and organic-like materials, four different contact materials were chosen to test, each representing varying levels of hardness and resistance: stone, wood, hide (fresh, raw, dry, tanned) and human skin. In addition, wood was used to support each of the different types of animal hides. Because of the location and distribution of the archaeological wear traces (on the lower/flat surface of the axe) it was decided that grinding and rubbing ochre on hide lying directly on the ground or suspended would not create enough resistance and tension.

In seven out of eleven experiments lard was used as an additive to create a paste, producing a more homogeneous mixture of grinded ochre. This was undertaken in order to replicate the archaeological wear traces, which resembled hide possibly with a more fatty additive. Adding lard to the ochre also served a more practical purpose in that it helped spread the ochre over and into the hide’s surface. While the main motion performed was scraping and grinding, in some cases, polished stone surfaces were used for the initial crushing of larger lumps of ochre before that same stone was used to grind and work the smaller pieces of ochre into the substrate. Duration of the experiments varied; the minimum working time was 30 min and the maximum 60 min. Each experimental polished stone surface was observed once after 30 min of activity and then again at the end: enabling the observation of wear development through time.

### pXRF methodology

Geochemical concentrations of lithic samples were analysed non-invasively with a Bruker S1 Titan portable X-fluorescence spectrometer (pXRF) with a 50 kV X-ray tube, SDD-detector and 8 mm spot-site. The acquisition time was 300 s, divided into two phases (Phase 1: 45 kV and 7.1 µA with TiAl filter; Phase 2: 15 kV and 17.45 µA without a filter). The reported results are mean values of 5–10 analyses per sample, quantified with the manufacturer’s Geochem Trace calibration using Compton ratio standardisation and fundamental parameters. The results were normalised to 100% apart from trace element concentrations reported as quantified, oxygen by stoichiometry. Hierarchical cluster analysis (Ward’s linkage; IBM SPSS 28 software) was run with the concentrations of K_2_O, CaO, TiO_2_, MnO, Fe_2_O_3_, ZnO, SrO, and ZrO_2_, demonstrated in previous studies to measure reliably by pXRF of unprocessed lithic surfaces in-air^49–56^. Elements that systematically showed concentration values close to the detection limit (< 50 ppm) were not considered for statistical tests.

### Petrographic analysis

Petrographic characteristics of the stones used were determined with the naked eye and using a Bresser ETD-201 stereo microscope with Bresser MicroCamSP 5.0 and a Zeiss Stemi 508 stereo microscope with Axiocam 508 colour.

### Morphometric analysis

Standard measurements were taken of axes (length, width at multiple locations, and maximum thickness) and plotted to explore the distribution and variation across the dataset. Exclusively complete (unfragmented) axes were plotted. The relevant boxplot was created using R 4.2.1^57^ and three packages in R: ggplot2^58^, data.table (v1.14.8^59^), and showtext (v.0.9-6^60^).

### GIS

Given the lack of authoritative ground survey data, the location of the trenches were established using the published hand drawn trench outlines^10,11^. However, the accuracy of the original maps is relative and their details vary by publication. These published maps were used in conjunction with available Lidar data for Latvia to situate the excavation. Each burial was then digitised as point data with associated burial numbers as attributes using ESRI ArcGIS Pro. Since an alphanumeric system, based on a 2 × 2 m square grid, was originally used to recover the settlement finds, the location of individual finds is approximate.

### Supplementary Information

Supplementary Information.

## Data Availability

All primary data produced as part of this study is available within the Supplementary Information.
