# Supplementary material for: Stone axes throw new light on Baltic stone age mortuary rites
Source: Sci Rep. 2024 Jul 13;14:16219. doi: 10.1038/s41598-024-66854-9 (PMC11246506; doi:10.1038/s41598-024-66854-9)
Supplement: Supplementary file 1 — Supplementary Information. [file 41598_2024_66854_MOESM1_ESM.docx]

# Supplementary Information

Supplementary Data S1: Axe technical report

The artefacts examined in this paper are called ‘axe’ (over ‘adze’). However, the term used does not necessarily reflect the original use or function of the artefact. Axe and adze are used interchangeably in the research area, and the choice is often more related to the specimen’s size than other morphological characteristics.

### Technology and morphology

Axes in the group 1 burials are morphologically and technologically similar. Their length is 103–111 mm, width 44–55 mm, thickness 15–20 mm and weight 139–173 g (Supplementary Table S1). The profile is flat, planar shape widening from the butt to the blade, and the cross-section is rectangular with one (upper) or more convex surface. The blade is evenly descending from one side in profile and curved or almost straight in planar view. In profile, the butt is rounded or straight and thin. The axes were finished from preforms by polishing which, while relatively good, is still uneven, and all three specimens have some of the unmodified surface visible. The axe from burial 32 (VI93:37) is the most completely polished, however there is damage at the blade end which is possibly modern. All display butts that are unpolished except from the axe in burial 59 which is partially polished.

In group 2, the axe from burial 211 (VI93:470) resembles the axes from group 1, but has different dimensions (79 x 35 x 25 mm, 102 g), a lenticular profile and thin butt. This specimen has a counterpart at the settlement site (no VI168:2338). The axe (VI93:674) from burial 233 has no parallels with other finds from Zvejnieki burals or settlement contexts. It is 46 x 22 x 13 mm in size and weighs 20 g, widening from the butt to the blade both in planar view and profile. It has a trapezoidal cross-section and steep faceted surfaces and sides with excellent and complete polishing; the blade is almost straight in planar view. Typologically, this axe resembles the so-called east or Russian Karelian axes.

The examined axes from the settlement areas include altogether 17 complete (but heavily damaged) specimens and 4 fragments (Supplementary Table S1). The length of the unfragmented specimens varies between 50–127 mm, the width at the butt is up to 44 mm and at the blade 35–64 mm, the thickness is 12–33 mm and weight, excluding one very large outlier, is 44–245 g. The axes from the settlement are morphologically more diverse than those from the burials, though a number (VI92:408, VI168:2682, also VI168:2328) appear to share similar morphology with the “flat-type” axes from burial group 1. In general, the settlement axes are straight or widen from the butt to the blade in planar view, the cross-sections are rectangular, lenticular or asymmetrical, with blades curved or almost straight; the butts are straight or rounded. In terms of coverage, the polishing varies from moderate to good. Five axes have blades at both ends, two specimens have a concave blade (gouge). In addition, there are two ‘ad hoc’ tools (no VI92:2, VI168:485) with minimal traces of modification apart from the blade.

Most of the axes from the settlement were found in the lower part (Zvejnieki I). A few differences between the form of axes could be identified between the different areas, with more double-axes and no gouges in the upper part. More broadly, the Zvejnieki axes correspond to the typologically varied group of artefacts known among the Mesolithic–Neolithic hunter-gatherer-fishers of the boreal zone^[e.g. 1,2]^. Research on these polished stone tools and their temporal settings has been sparse in Latvian archaeology ^[3,4,5]^. Based on other areas in boreal north-east Europe, axes with similarly simple morphologies and often only partial polishing were manufactured over thousands of years. Thus, even if the axes in group 1 represent a type of production that began in the Mesolithic, in general, the axe forms present at Zvejnieki do not display significant chronologically diagnostic features. The main exception is VI93:674 from burial 233, which finds morphological parallels further north in the East or Russian Karelian tool industry centred in the Lake Onega region, giving it an approximate 4^th^ millennium cal. BC date^[6]^.

### Provenance

To assess whether any variation exists in the geological sources and whether the provenance of the raw material can be determined as local or imported, altogether 26 axes were analysed for petrography and geochemical composition. The sample includes all 5 axes found in burials and 21 specimens from Zvejnieki I and II settlements, including the 17 axes that were sub-sampled for microwear analysis. To test the local origin hypothesis, an additional 16 geological rock reference samples were collected from the Zvejnieki drumlinoid ‘cap’ area and the adjacent Košķele drumlin (both located in the central part of the Burtnieks drumlin field in the Lake Burtnieks area). Petrographic determinations of the archaeological and reference samples are given in Supplementary Table S4. In group 1, all axes are porphyrite (no VI93:37 and VI93:66 uralite hornblende porphyrite and no VI93:59 Tammela uralite porphyrite), while in group 2, no VI93:470 is microgabbro and no VI93:674 amphibolite. At the settlement site the most common stones are diabase (6) and (Tammela) hornblende porphyrite (5), followed by amphibolite (3), only in the upper settlement, gneiss (3), microgabbro (olivine gabbro) (2), with two individual axes of basalt and phyllite. The flat axes are most often made of porphyrite and diabase but also of other rock types; gouges and double axes are made of porphyrite, diabase, amphibolite and basalt. The ‘ad hoc’ tools are made from gneiss. The most common rocks in the geological reference samples are gneiss (7), amphibolite (5), but include also porphyrite (porphyritic hornblende and epiodotized feldspar porphyrite) (2), diabase (1) and migmatite (1).

Geochemical concentrations of 42 lithic samples (26 axes, 16 reference samples) were analysed non-invasively on clean surface areas with a portable X-fluorescence spectrometer (pXRF) (Supplementary Table S4). The cluster analysis dendrogram of the geochemical data indicates five clusters (I–V) of compositionally related samples, and additional outliers. Geological reference samples of local rock materials are present in Clusters I, III and V; Clusters II and IV contain only axes (Supplementary Table S5).

The petrography of axes and the geological reference samples shows overlap, including amphibolite, gneiss, also porphyrite and diabase. Given that the analysed reference samples cluster together with the axes (burial and settlement) it seems likely that the material used for most of the studied axes was available locally and sourced close to the site. However, in geological terms the rocks are not of local origin, since the surface of the bedrock at Zvejnieki area consists mainly of sandstones and siltstones of the Middle Devonian Burtnieks Series. All crystalline rocks were torn out of the bedrock in the area of present-day Finland and Sweden and carried by the glacier during the last Ice Age to the Burtnieki drumlin field. The varied origins are reflected in both reference samples and raw materials used in axe production. Artefact nos. VI92:51, VI92:261, VI93:674 and VI168:2594 are not compositionally related to the other analysed materials, suggesting that they were either made of local raw materials not included in the reference samples analysed, or were imported. The latter cannot be determined, due to the generally non-diagnostic morphological features of the axes, apart from VI93:674 from burial 233. This small axe is made of greenish amphibolite, visually resembling the so-called metatuff found in a restricted area in the Lake Onega region and used extensively there in production of Russian Karelian artefacts^[6]^, representing a long distance import. Otherwise, sourcing is best characterised as localised and opportunistic, with no clear patterns between geology used and the broadly defined axe types. The only exception may be the flat axes VI93:37, VI93:59 and VI93:66 from burial group 1, which are uralite (hornblende) porphyrite (Clusters IV and V; cf. also flat axes VI92:164, VI168:2682 and VI168:2328 of porphyritic hornblende in these clusters). Their similar raw material indicates that the stone these axes were made from possibly came from the same (local) source. Additionally, their technological and morphological similarity, as well as deposition in spatially adjacent graves with similar burial customs, suggests group 1 axes were made and deposited as part of funerary activity which was temporally brief compared to the cemetery’s entire period of use.

| **Artefact number** | **Type** | **Weight (g)** | **Lenght (mm)** | **Width/butt (mm)** | **Width/middle (mm)** | **Width/blade (mm)** | **Thickness (max, mm)** |
| --- | --- | --- | --- | --- | --- | --- | --- |
| **Burials** | | | | | | | |
| VI93:37 | Axe | 172.7 | 110.9 | 20.8 | 43.8 | 43.8 | 20 |
| VI93:59 | Axe | 138.8 | 103.3 | 36.2 | 46.9 | 49.4 | 15.4 |
| VI93:66 | Axe | 154 | 107.5 | 41.3 | 51.8 | 54.6 | 15.7 |
| VI93:674 | Axe (Russian-Karelian) | 20.4 | 46.1 | 11.3 | 20.6 | 22.3 | 13.4 |
| VI93:470 | Axe | 102.2 | 78.1 | 21.2 | 34.4 | 34.5 | 25 |
| **Settlement** | | | | | | | |
| VI92:249 | Double axe (even+gouge) | 98 | 65.8 | 42.4 | 43.5 | 40.5 | 21.8 |
| VI92:164 | Axe | 56.3 | 60.1 | 16 | 36.1 | 42.9 | 14.3 |
| VI92:305 | Axe | 76.5 | 57.7 | 41.8 | 45.9 | 41.7 | 16.5 |
| VI92:278 | Double axe (even+even) | 112.2 | 82.8 | 34.4 | 46.6 | 47 | 15.8 |
| VI92:261 | Axe (gouge?), central fragment | 88.8 | 74.7 | 39.5 | 39.4 | 40.3 | 18.6 |
| VI92:408 | Axe | 196.1 | 98.5 | 44.4 | 54.7 | 56.4 | 19 |
| VI92:445 | Axe (gouge) | 227 | 101.5 | 32.1 | 45.8 | 46.8 | 26.1 |
| VI92:51 | Axe, butt fragment | 102.3 | 59.7 | 41.8 | 46.1 | 41.9 | 19.8 |
| VI92:111 | Axe | 186.7 | 99 | 29.5 | 36.1 | 40.3 | 26.5 |
| VI92:407 | Axe | 194.2 | 90 | 29.5 | 47.1 | 53.3 | 23.9 |
| VI92:529 | Axe, side fragment | 120.8 | 96.7 | 26.2 | 38.9 | 36.9 | 19.3 |
| VI92:136 | Axe, butt fragment | 37.5 | 43.7 | 28.3 | 33.7 | 36.1 | 15.8 |
| VI92:2 | Axe (“ad hoc”) | 245.3 | 116.2 | 36 | 44.9 | 35.7 | 26.2 |
| VI92:90 | Axe | 43.9 | 50.2 | 28.3 | 36 | 37.8 | 12.7 |
| VI92:97 | Axe | 99.2 | 82.4 | 31.5 | 43.5 | 44.9 | 16.8 |
| VI168:799 | Axe | 77.8 | 64 | 22.8 | 37.8 | 45.4 | 16.8 |
| VI168:2594 | Double axe (even+even) | 117.2 | 87 | 2 | 33.3 | 35.1 | 23.9 |
| VI168:485 | Double axe (“ad hoc”, even+even) | 383.2 | 127.1 | 38.7 | 70.4 | 64.2 | 32.5 |
| VI168:2682 | Double axe (even+even) | 241.1 | 110.4 | 35.8 | 54.7 | 54.2 | 22.8 |
| VI168:2338 | Axe | 141.9 | 77.9 | 31.7 | 38.2 | 37.6 | 25.6 |
| VI168:2328 | Axe | 150.2 | 88.9 | 41.3 | 49.2 | 52.4 | 19.7 |

Supplementary Table S1: Weights and measurements of axes from Zvejnieki burials and settlement

| **Sample** | **Contact material** | **Additive** | **Activity** | **Motion** | **Duration** | **Wear** |
| --- | --- | --- | --- | --- | --- | --- |
| A5 | Stone |  | Crushing, grinding, and rubbing | Back/forth | 60 mins | 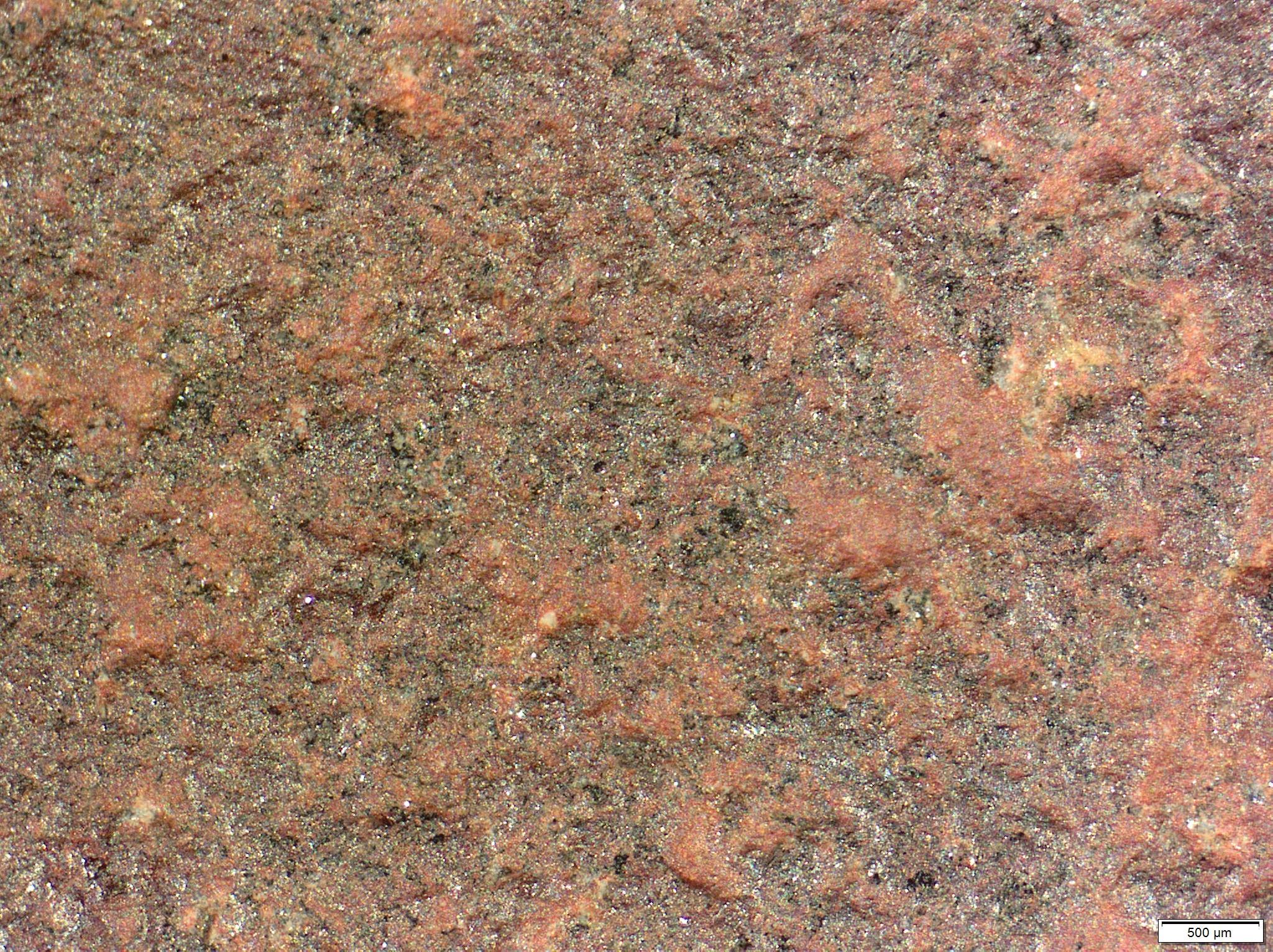 |
| A4 | Wood |  | Grinding and rubbing | Back/forth | 50 mins | 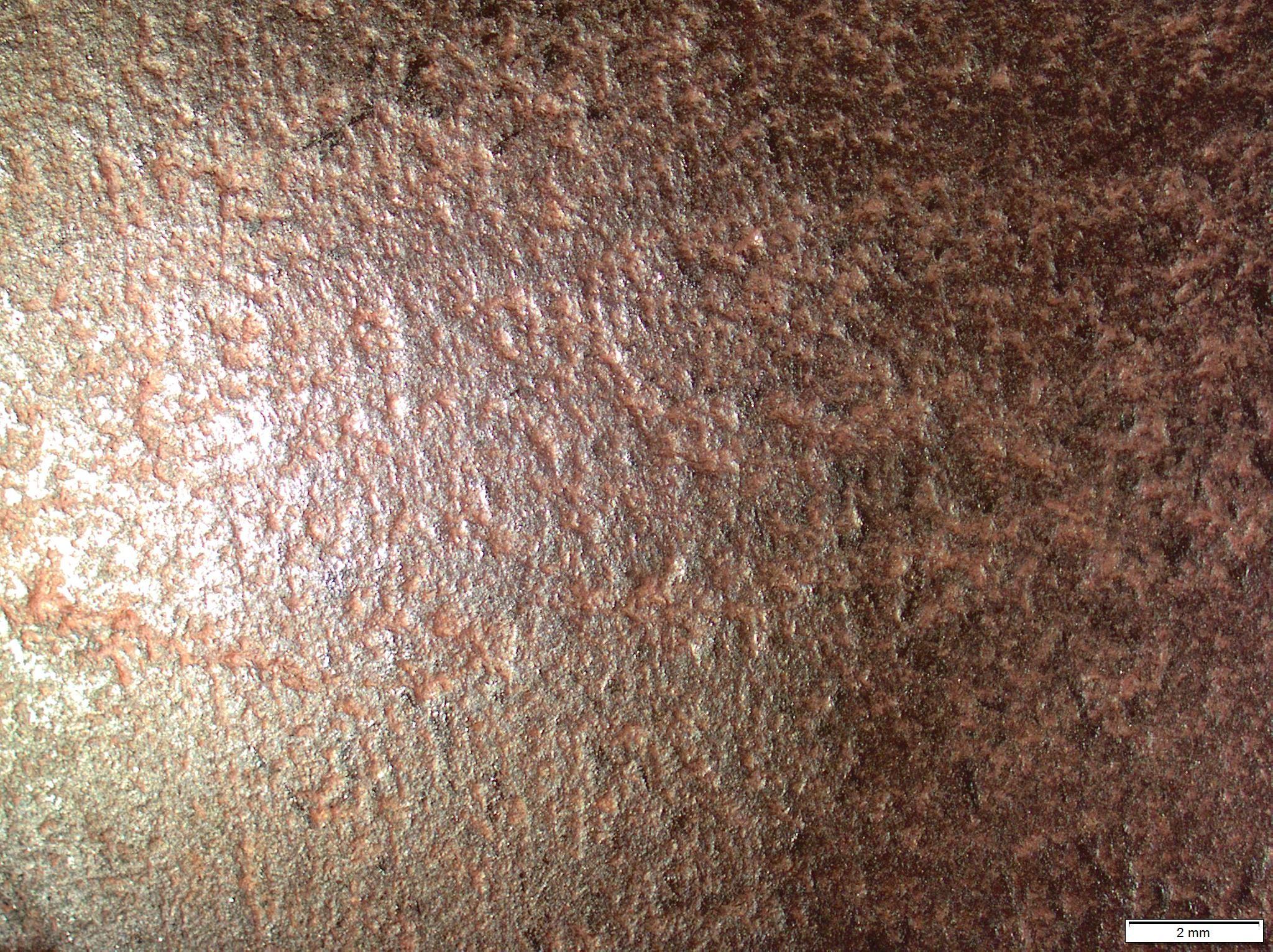 |
| A2 | Dry hide on wood |  | Rubbing | Back/forth | 30mins | 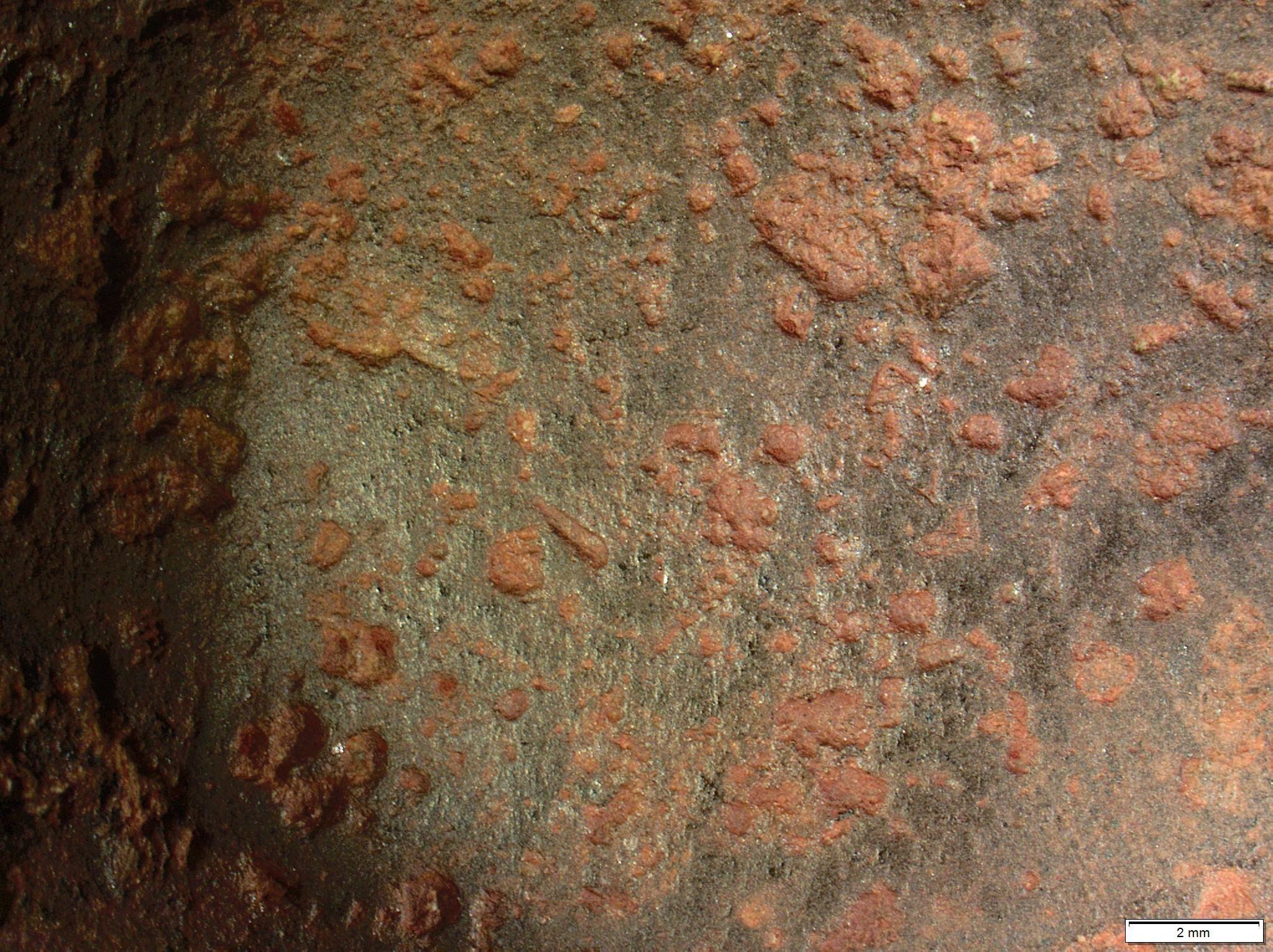 |
| A9 | Rawhide on wood | Lard | Rubbing | Back/forth | 60 mins | 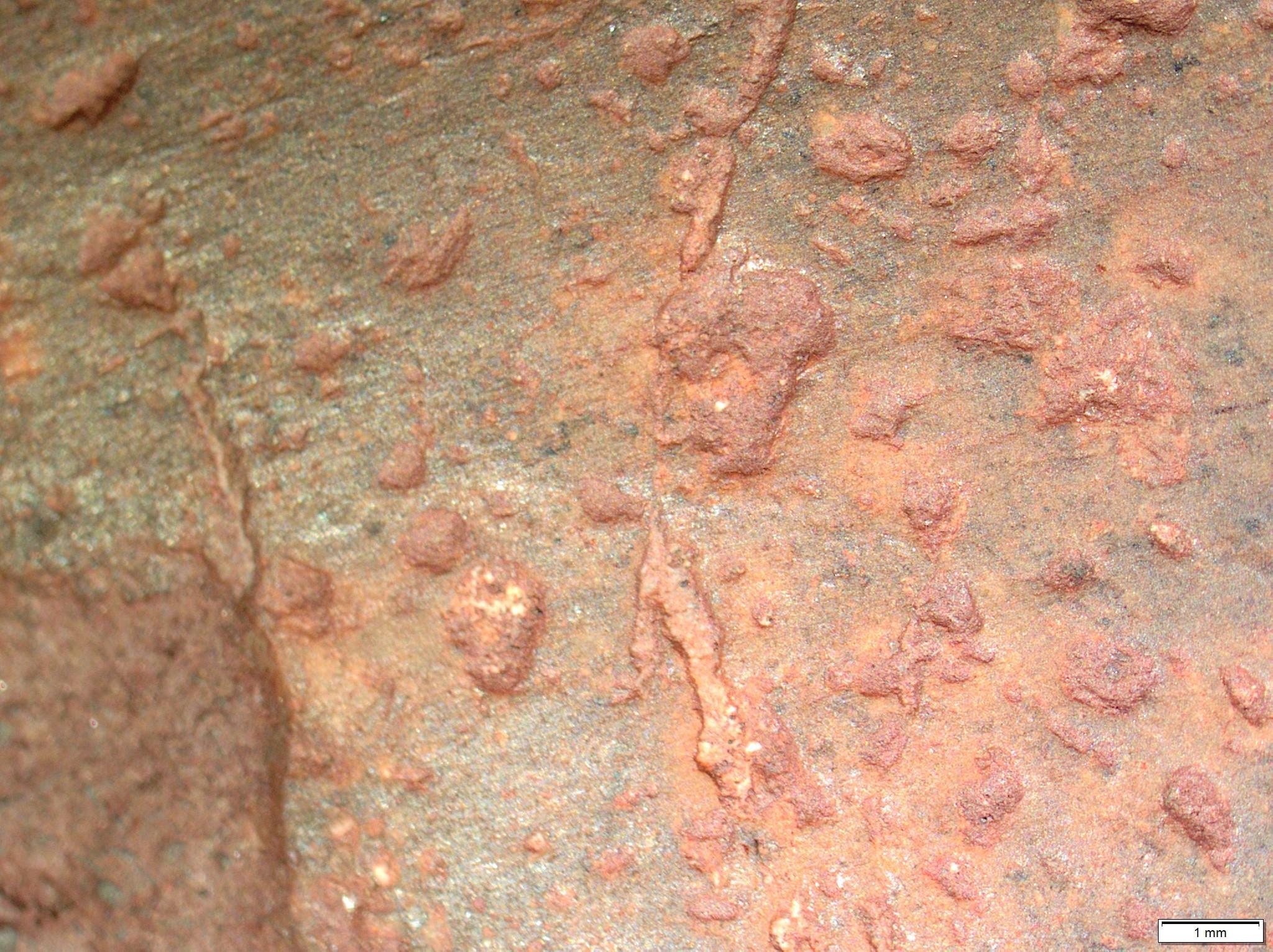 |
| A10 | Dry hide on wood |  | Rubbing | Back/forth | 30 mins | 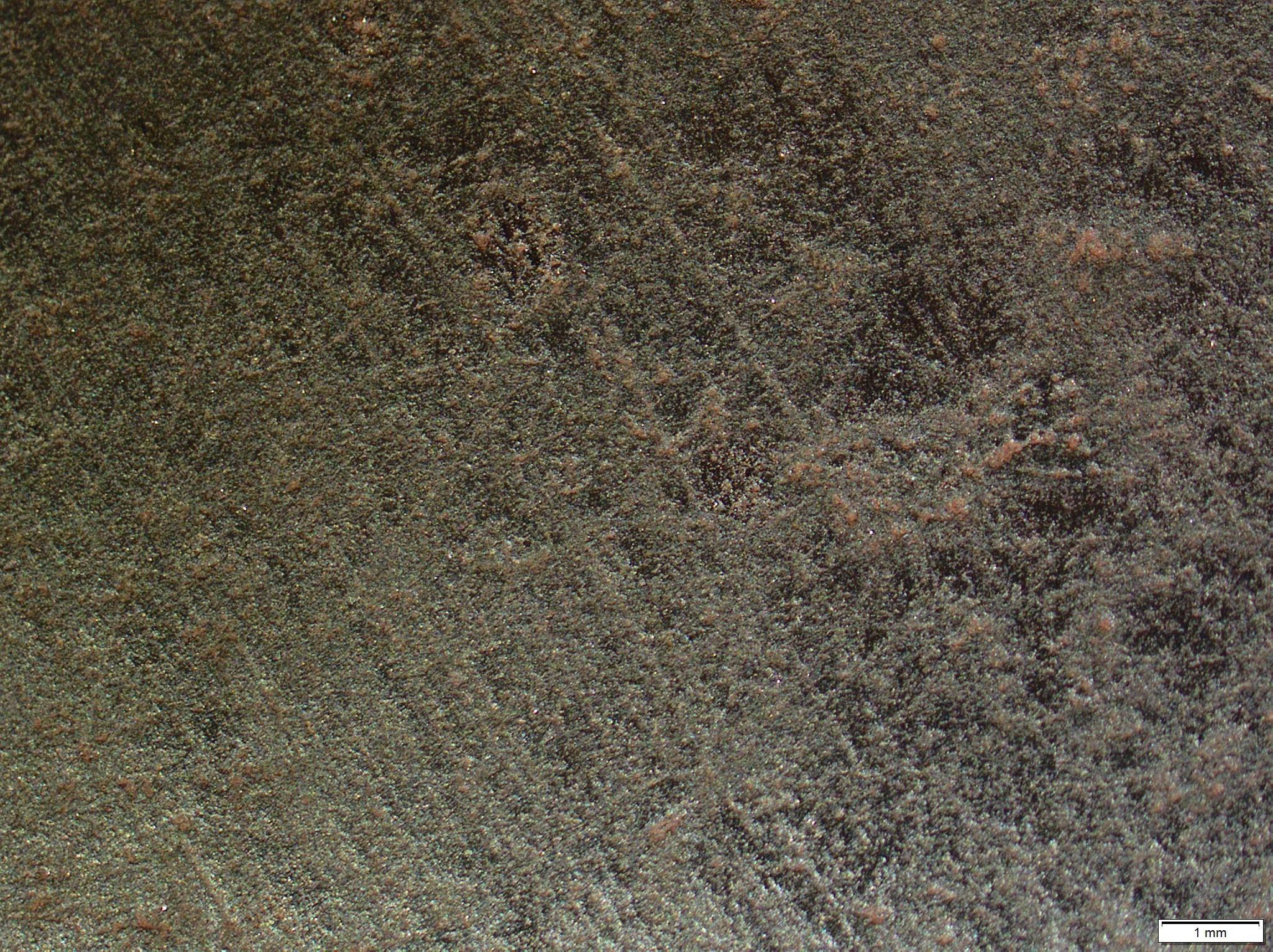 |
| A7 | Rawhide on wood | Lard | Rubbing | Back/forth | 33 mins | 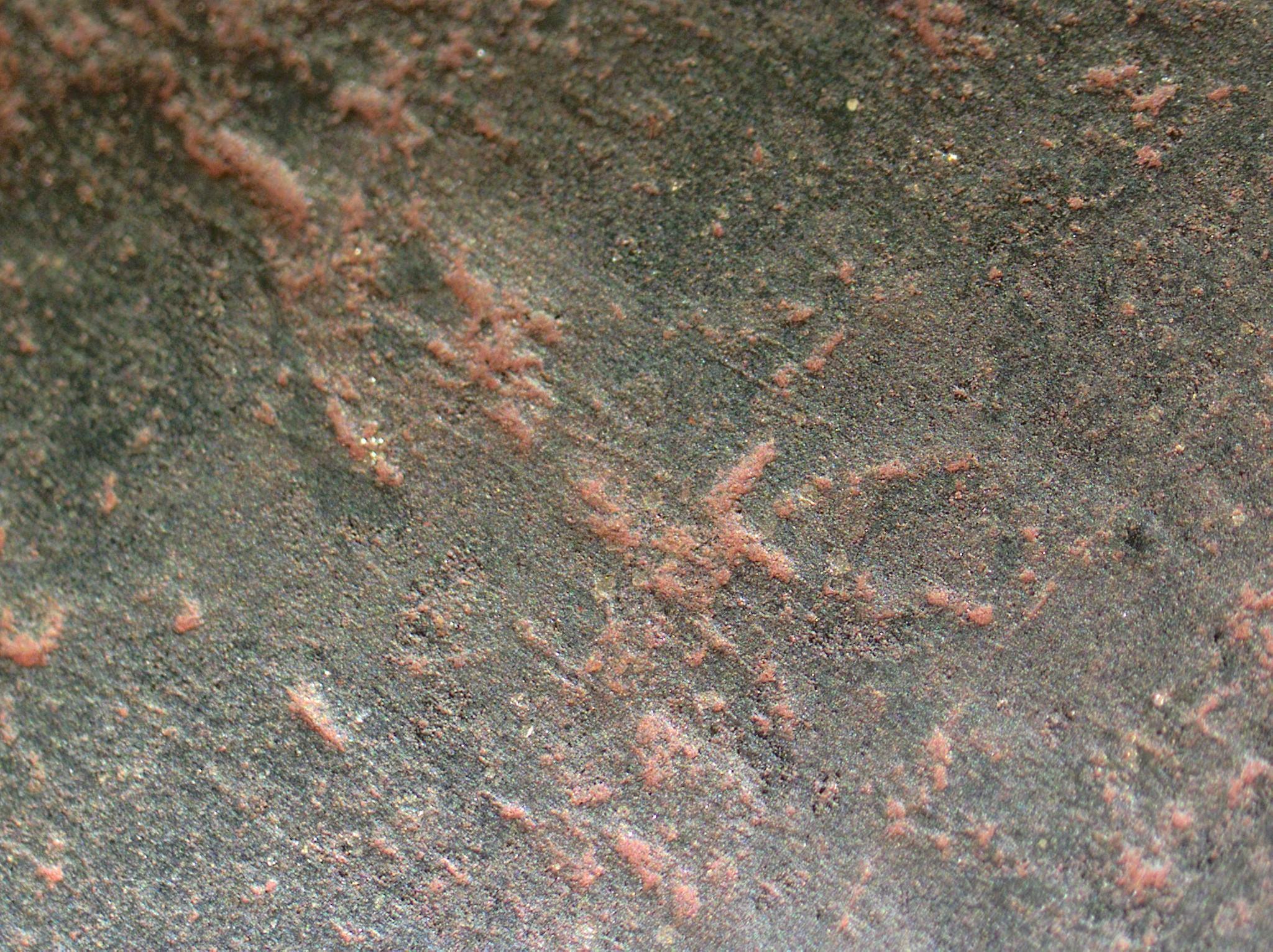 |
| A3 | Tanned hide (roe deer) on wood | Lard | Rubbing | Back/forth | 30 mins | 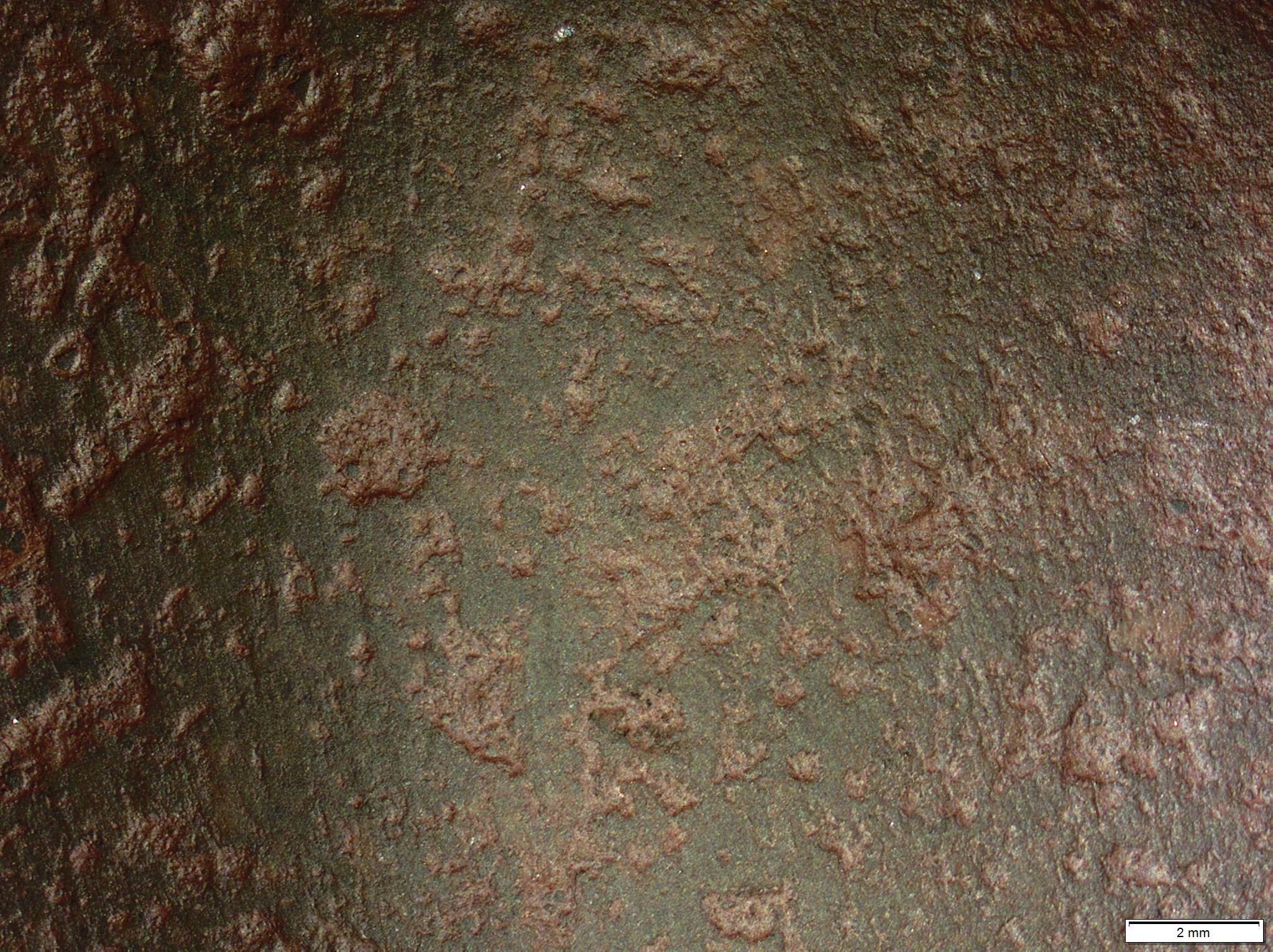 |
| A6a | Tanned (grape seed oil) and smoked hide (fallow deer) on wood | Lard | Rubbing | Back/forth | 30 mins | 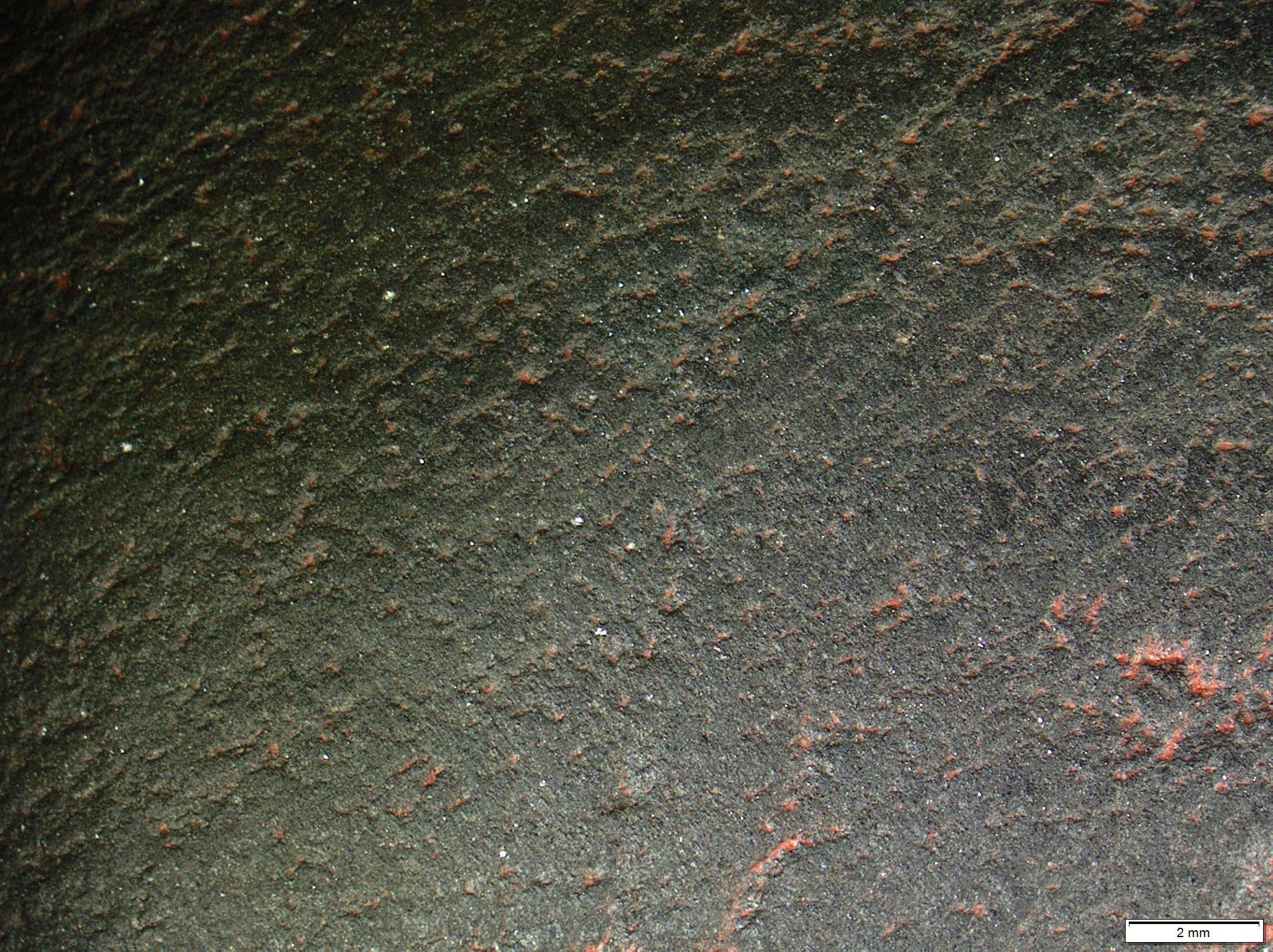 |
| A8 | Tanned and smoked hide (white-tailed deer) with brains on wood | Lard | Rubbing | Back/forth | 30 mins | 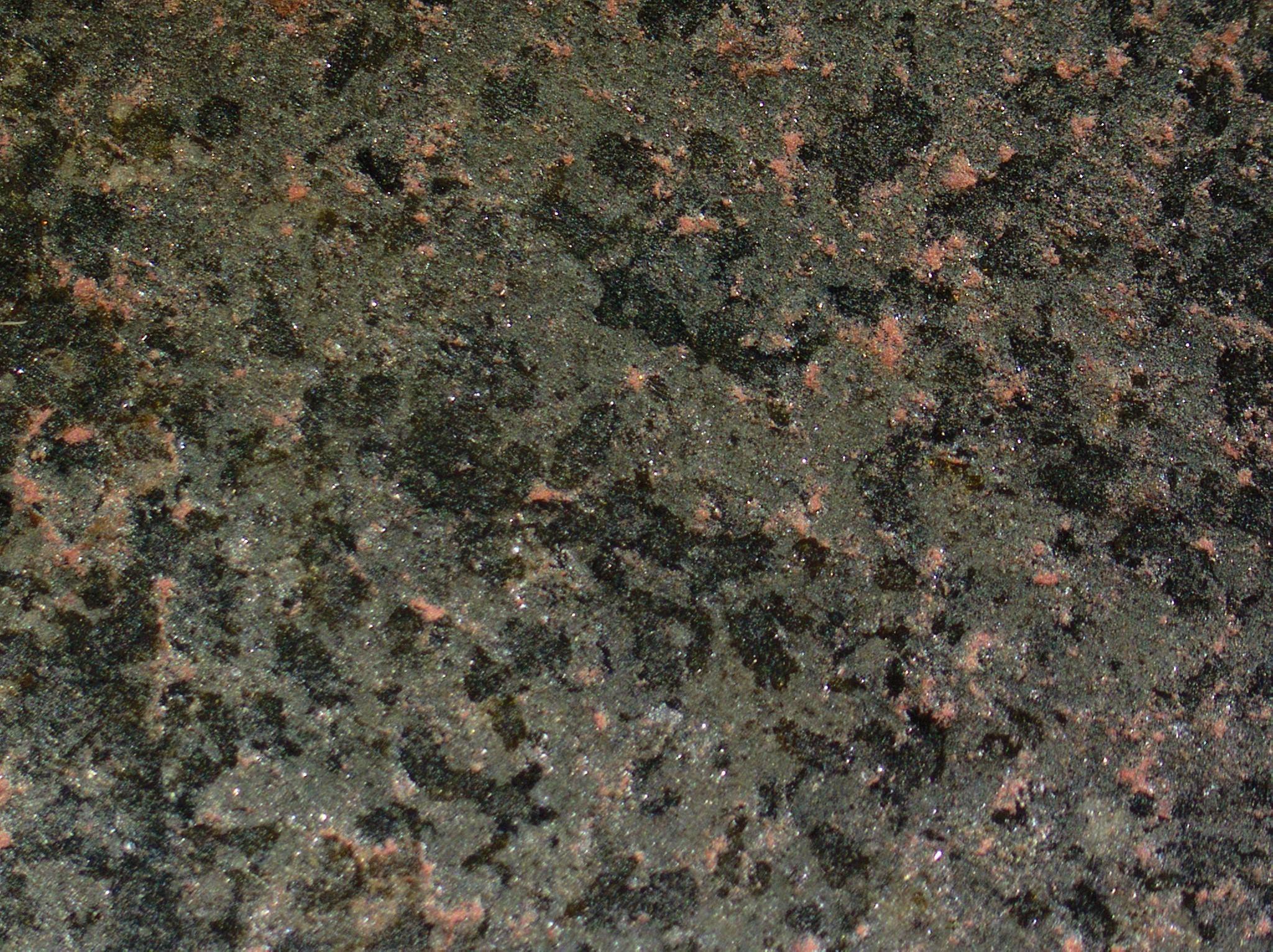 |
| A1 | Human skin | Lard | Rubbing | Back/forth | 60 mins | 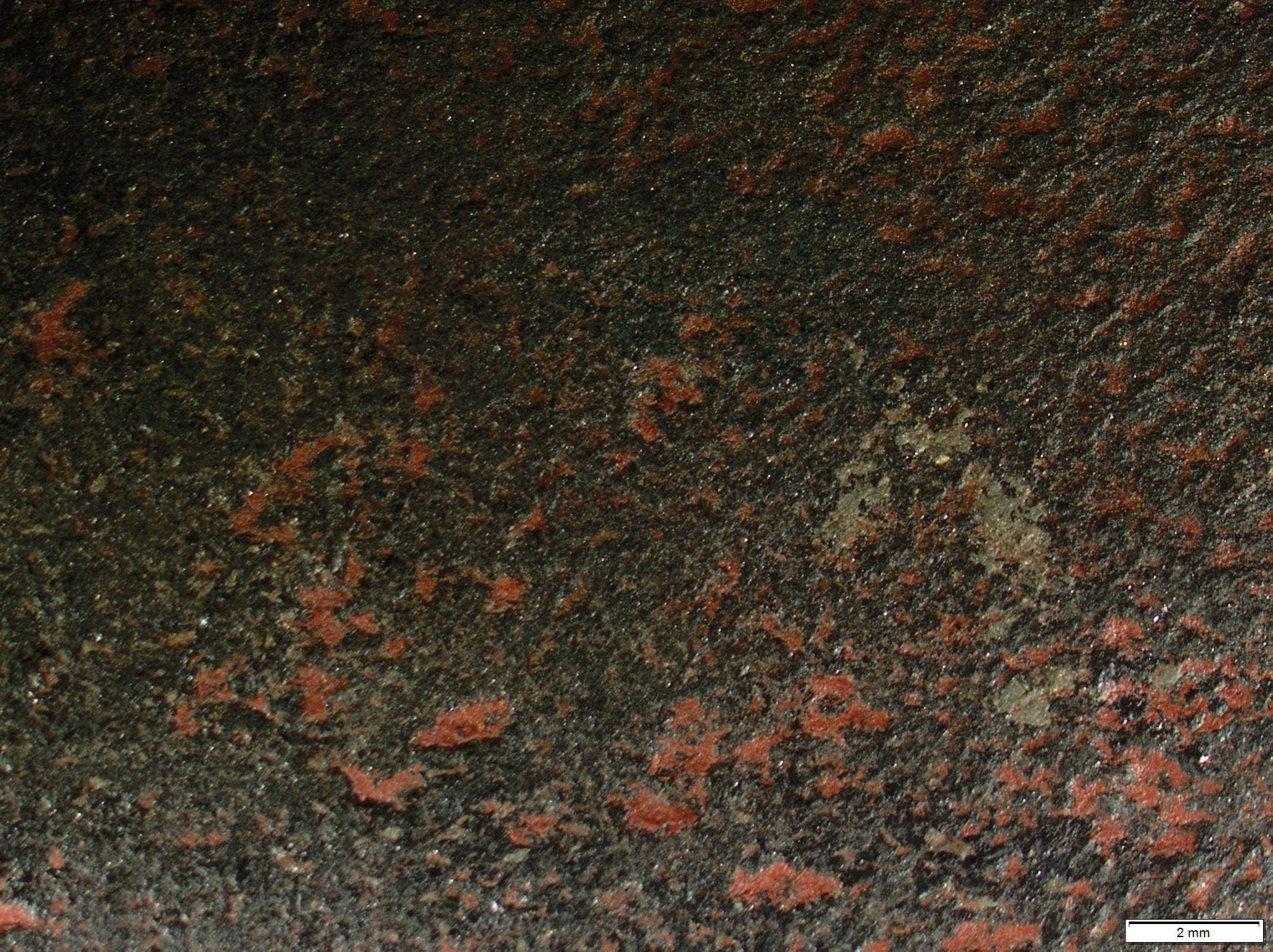 |
| A6b | Fresh animal skin (fallow deer) on wood (both sides) | Lard | Rubbing | Back/forth | 60 mins | 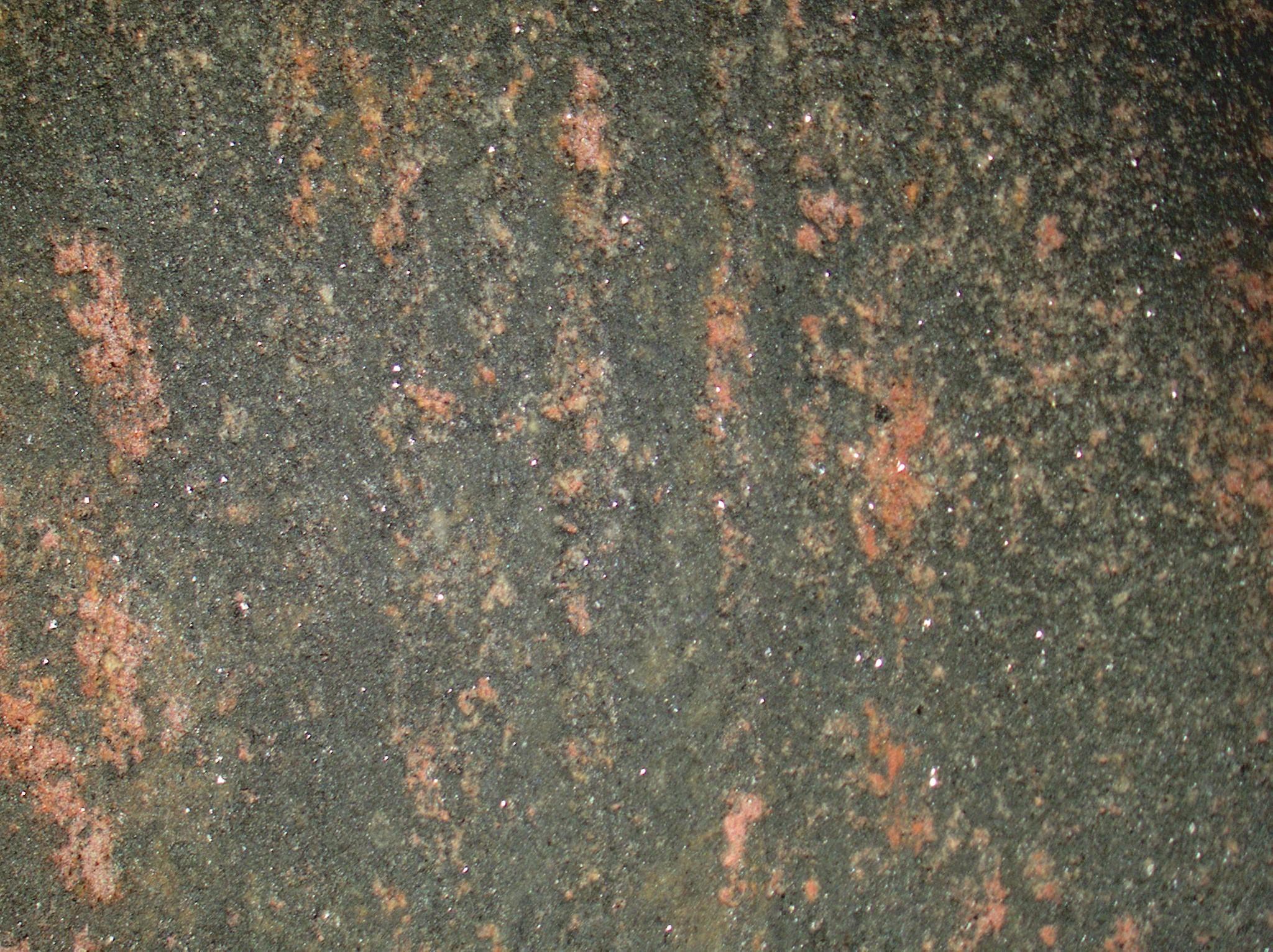 |

**Supplementary Table S2:** Experimentally produced reference collection of ochre, hide and lard-working wear traces using polished stone surfaces (Porphyritic Trachyte, Hornfels and Cordierite Hornfels)

| **Context** | **Artefact number** | **Description of wear** | **Interpretation** |
| --- | --- | --- | --- |
| Axes from burial contexts | | | |
| Burial 32 | VI93:37 | No visible traces | Not used |
| Burial 51 | VI93:59 | No visible traces | Not used |
| Burial 57 | VI93:66 | Lower areas: rough to smooth texture, granular topography, open to half-tight linkage  Upper areas: bright and smooth texture with flat topography | Processing ochre and animal material |
| Burial 211 | VI93:470 | No visible traces | Not used |
| Burial 233 | VI93:674 | No visible traces | Not used |
| Axes from settlement contexts | | | |
| Zvejnieki I, area A3 | VI92:408 | Not diagnostic | Used |
| Zvejnieki I, area D | VI92:445 | Not diagnostic | Used |
| Zvejnieki I, area A3 | VI92:407 | Traces of wear, but alterated | Used |
| Zvejnieki I, area A | VI92:51 | Traces of wear, but alterated | Used |
| Zvejnieki I, area A1 | VI92:111 | Traces of wear, but alterated | Used |
| Zvejnieki II, area IV | VI168:485 | Rounded, altered, soil sheen | Used |
| Zvejnieki II | VI168:799 | Step trace termination, alterated | Used, for short period of time |
| Zvejnieki II, area XVII | VI168:2594 | Step trace termination, small edge rounding | Used, for short period of time |
| Zvejnieki II, area XVII | VI168:2682 | Step and step traces | Used |
| Zvejnieki II, area XVI | VI168:2328 | Edge and upper surfaces mainly missing due to use, lower surface intact, soil sheen, micro not diagnostic | Hard material, longer period of time |
| Zvejnieki II, area XVI | VI168:2338 | Upper surface mainly missing due to use, lower surface intact, micro not diagnostic | Hard material, longer period of time |

**Supplementary Table S3:** Microwear analysis of all axes from Zvejnieki burials and settlement (sub-sample)


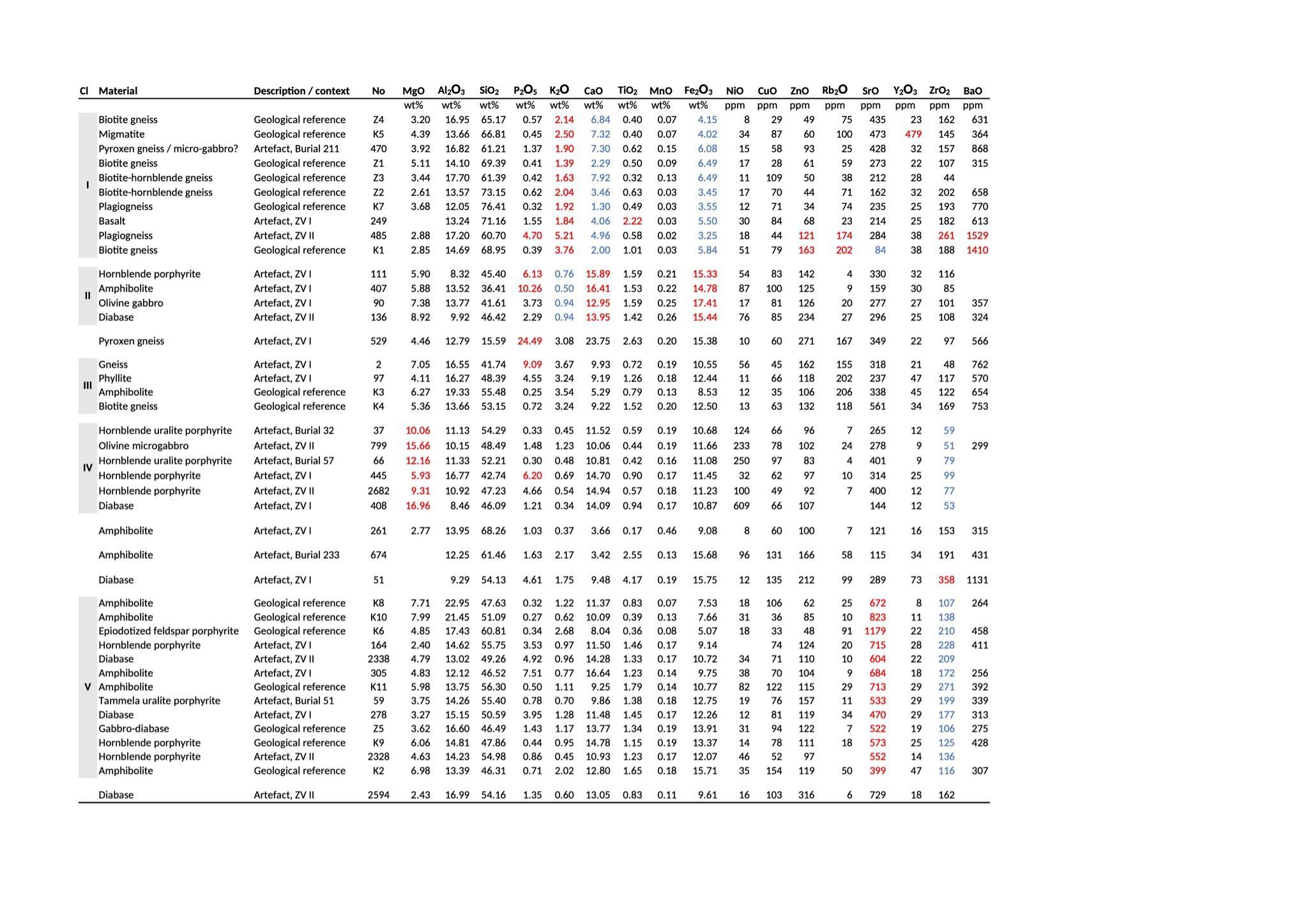


**Supplementary Table S4:** pXRF measured chemical compositions of lithic artefacts and potential source rocks, order and cluster assignments by CA dendrogram. Five clusters (I–V) of compositionally related samples are indicated, with additional outliers. In addition to artefacts, geological reference samples of local rock materials are present in Clusters I, III and V. Cluster I includes seven geological reference samples and three artefacts (nos. VI92:249, VI93:470 and VI168:485) mostly of gneiss characterised with CaO concentrations <8 wt%, Fe_2_O_3_ <6.5 wt% and NiO, CuO, ZnO, Rb_2_O values ≤110 ppm (apart from artefact 485 and reference K1 with ZnO and Rb_2_O values <200 ppm). Cluster II is a varied group of hornblende-amphibolite-gabbro-diabase artefacts, nos. VI92:90, VI92:111, VI92:136 and VI92:407, which show higher P_2_O_3_, CaO, Fe_2_O_3_ values (≤10.3 wt% ≤16.4 wt% and ≤17.4 wt%, respectively) compared to Cluster I. Artefact no VI92:529 is discriminated from Cluster II by its P_2_O_3_ and CaO values beyond the group’s ranges. Cluster III includes two artefacts (nos. VI92:2 and VI92:97) and two reference rocks which present higher K_2_O and Rb_2_O values compared to the other groups. Cluster IV members are mostly porphyrite artefacts (nos. VI93:37, VI93:66, VI92:408, VI92:445, VI168:799 and VI168:2682) displaying the highest range of MgO values (ca. 6–17 wt%) showing comparatively high CaO, Fe_2_O_3_ and NiO and low ZrO_2_ concentrations. In Cluster V, rock reference samples K2, K6, K8–11 and Z5 share general geochemical compositional patterns with six artefacts (nos. VI93:59, VI92:164, VI92:278, VI92:305, VI168:2328 and VI168:2338) determined as amphibolite-porphyrite-diabase rocks, characterised with relatively high CaO at 8–17 wt% and SrO at ca. 400–1200 ppm.


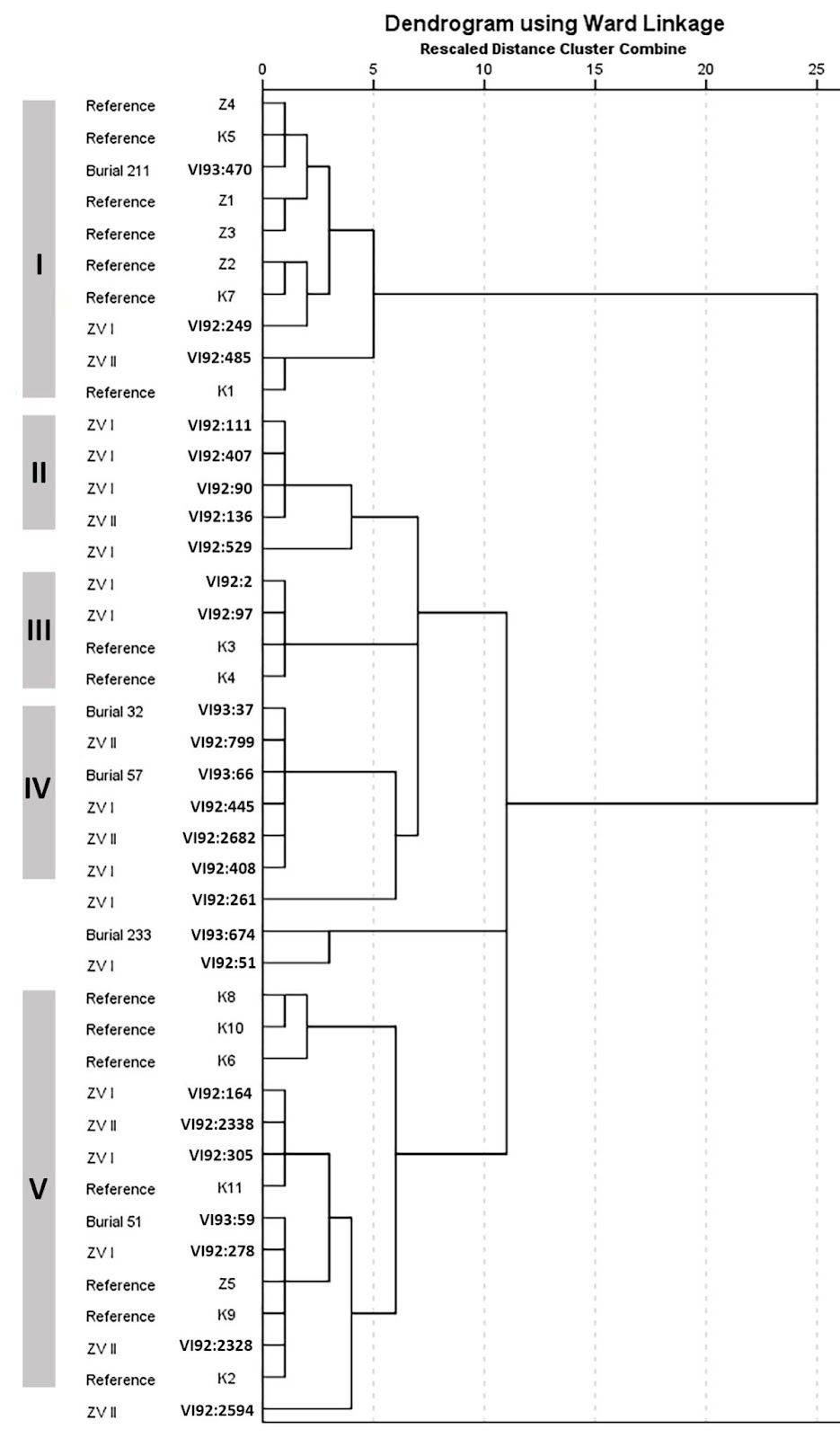


**Supplementary Table S5:** pXRF measured chemical compositions of lithic artefacts and potential source rocks, order and cluster assignments by CA dendrogram (K_2_O, CaO, TiO_2_, MnO, Fe_2_O_3_, ZnO, SrO, and ZrO_2_ concentrations). ZVI (Zvejnieki I), ZVII (Zvejnieki II), Reference (non-archaeological stone, locally sourced).
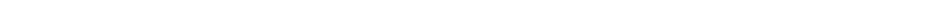


References

1. Ailio, J. *Die Steinzeitlichen Wohnplatzfunde in Finland I-II* (Suomen Muinaismuistoyhdistys, 1909).

2. Pankrushev, G. A. *Mezolit i neolit Karelii, ch. 1 mezolit* (Nauka, 1978).

3. Šturms, E. *Die steinzeitlichen Kulturen des Baltikums (Antiquitas, 3.9)* (Rudolf Habelt, 1970).

4. Bīrons, A. *et al.* (eds) *Latvijas PSR arheoloģija* (Zinātne, 1974).

5. Graudonis, J. *et al.* (eds) *Latvijas senākā vesture. 9. G.t. pr.Kr. – 1200 G* (Latvijas vēstures institūta apgāds, 2001).

6. Tarasov, A. & Nordqvist, K. Made for exchange: the Russian Karelian lithic industry and hunter-gatherer exchange networks in prehistoric north-eastern Europe. *Antiquity* **96**(385), 34–50 (2022).
